# Advancing understanding of human variability through toxicokinetic modeling, in vitro-in vivo extrapolation, and new approach methodologies

**DOI:** 10.1186/s40246-024-00691-9

**Published:** 2024-11-21

**Authors:** Anna Kreutz, Xiaoqing Chang, Helena T. Hogberg, Barbara A. Wetmore

**Affiliations:** 1 Inotiv, 601 Keystone Park Drive, Suite 200, Morrisville, NC 27560 USA; 2 Oak Ridge Institute for Science and Education, Oak Ridge, TN 37830 USA; 3 NIH/NIEHS/DTT/NICEATM, Research Triangle Park, NC 27560 USA; 4 Office of Research and Development, Center for Computational Toxicology and Exposure, US Environmental Protection Agency, Research Triangle Park, NC 27711 USA

**Keywords:** Toxicokinetic variability, Physiologically based toxicokinetic model, In vitro-in vivo extrapolation, New approach methodologies, Life-stage

## Abstract

The merging of physiology and toxicokinetics, or pharmacokinetics, with computational modeling to characterize dosimetry has led to major advances for both the chemical and pharmaceutical research arenas. Driven by the mutual need to estimate internal exposures where in vivo data generation was simply not possible, the application of toxicokinetic modeling has grown exponentially in the past 30 years. In toxicology the need has been the derivation of quantitative estimates of toxicokinetic and toxicodynamic variability to evaluate the suitability of the tenfold uncertainty factor employed in risk assessment decision-making. Consideration of a host of physiologic, ontogenetic, genetic, and exposure factors are all required for comprehensive characterization. Fortunately, the underlying framework of physiologically based toxicokinetic models can accommodate these inputs, in addition to being amenable to capturing time-varying dynamics. Meanwhile, international interest in advancing new approach methodologies has fueled the generation of in vitro toxicity and toxicokinetic data that can be applied in in vitro-in vivo extrapolation approaches to provide human-specific risk-based information for historically data-poor chemicals. This review will provide a brief introduction to the structure and evolution of toxicokinetic and physiologically based toxicokinetic models as they advanced to incorporate variability and a wide range of complex exposure scenarios. This will be followed by a state of the science update describing current and emerging experimental and modeling strategies for population and life-stage variability, including the increasing application of in vitro-in vivo extrapolation with physiologically based toxicokinetic models in pharmaceutical and chemical safety research. The review will conclude with case study examples demonstrating novel applications of physiologically based toxicokinetic modeling and an update on its applications for regulatory decision-making. Physiologically based toxicokinetic modeling provides a sound framework for variability evaluation in chemical risk assessment.

## Introduction

Toxicokinetics (TK) is the study of xenobiotic fate in an organism, which requires the consideration of absorption, distribution, metabolism, and excretion (ADME) [1]. Alternatively, toxicodynamics (TD) describes the study of the effects a chemical may have on an organism, which in turn will require distinct considerations from TK [2]. Simply put, TK reflects what the body does to a chemical; TD reflects what the chemical does to the body.

A significant challenge worldwide in chemical or drug safety evaluations is understanding the extent and consequences of interindividual and population variability [3, 4]. Such variability can stem from differences in TK that lead to differing internal concentrations despite equivalent external exposure levels; or differences in TD, represented by differing susceptibilities to an adverse effect [5]. These research areas (i.e., TK and TD) are analogous to pharmacokinetics (PK) and pharmacodynamics (PD) as applied to pharmaceuticals, but with broader consideration of a wider range of exposures, dose–response, and effects or adverse outcomes that are important in toxicology during chemical risk assessment [6]. As such, the majority of the text, except when capturing historical PK concepts, will use the phrases TK and TD to describe TK/PK and TD/PD concepts. Regardless of which space is being considered, variability assessment requires distinct strategies for adequate consideration in human health risk assessment and has been an active area of research and discussion for well over 30 years [3–5, 7–9].

**Fig. 1:**
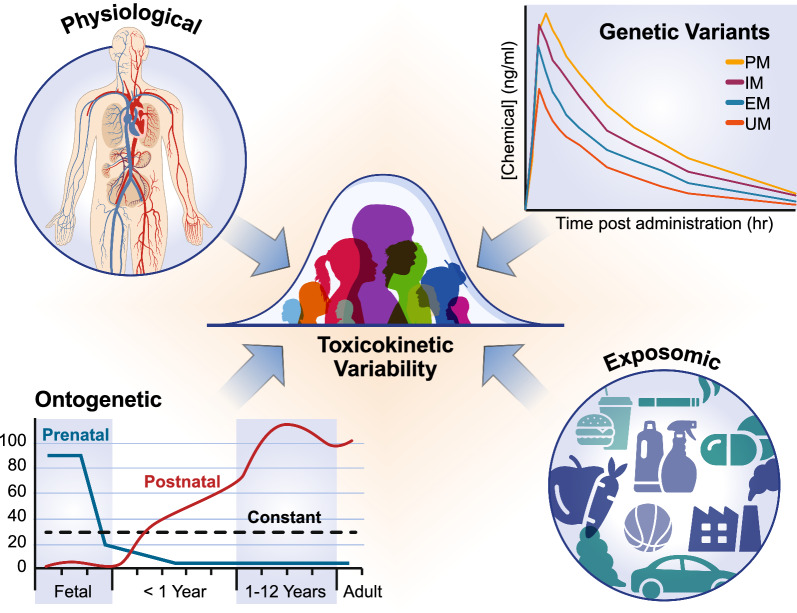
Drivers of toxicokinetic (TK) variability. Physiological drivers include factors such as blood flows. Genetic factors include polymorphisms in enzymes such as CYP2D6, which results in varying rates of compound metabolism by poor metabolizers (P M), intermediate metabolizers (IM), extensive metabolizers (EM), and ultrarapid metabolizers (UM). Ontogenetic factors include changes in CYP expression over the course of pre and postnatal development. Adapted from “Fig. 1: Key life stages” from “Guidance on the risk assessment of substances present in food intended for infants below 16 weeks of age,” by EFSA Scientific Committee, used under CC BY-ND 4.0 [20]. Exposomic drivers include diet, lifestyle, environmental justice, and exposure/co-exposures

Of particular note is the vast amount of knowledge gained regarding differences that contribute to TK variability, which include physiology, genetics, ontogenetics, and exposomics as depicted in Fig. 1. Data from genomics technologies have shown that numerous polymorphisms exist across enzymes and biological targets, and how these vary by ethnicity and life-stage [10, 11]. A concerted effort fueled by the recognition of developmental changes in enzymes has provided a rich source of Phase I and II metabolic enzyme abundance data across life-stages [12–15]. The subsequent emergence of targeted proteomics as a tool to monitor enzyme and transporter abundance has further expanded the ability to evaluate differences in distribution and metabolism [16, 17]. Recent progress in understanding xenobiotic-microbiome interactions is poised to inform lines of inquiry regarding impacts on chemical biotransformation, fate, and potential interactions that will contribute to variability [18, 19]. Population variability characterization remains a highly active research area.

In parallel, the toxicology community has made significant strides in developing new approach methodologies (NAMs) to evaluate chemical safety. Increased consideration of alternative testing strategies that incorporate experimental human in vitro methods and in silico modeling approaches has sparked substantial investment in industry, academic, and regulatory sectors with the aim of incorporating such models and systems into hazard identification [21–23]. NAMs allow for the interrogation of mechanistic pathways or endpoint-specific effects that may provide insight into chemical modes of action. Moreover, NAMs can be adapted for high-throughput screening and can circumvent the need for interspecies extrapolation by using human-specific in vitro models [24, 25]. While not designed to provide a full replacement for in vivo studies, these NAMs are beginning to be applied to inform certain decision contexts (e.g., prioritization) and even waive in vivo studies given sufficient supporting evidence, as they continue to mature [26, 27].

To harness the advantages of NAMs for human health risk assessment, assay readouts must be translated into in vivo metrics. This can be achieved through in vitro-in vivo extrapolation (IVIVE). Physiologically based toxicokinetic (PBTK) frameworks are particularly well suited for IVIVE by bridging the gap between in vitro and in vivo TK both qualitatively and quantitatively [28]. These PBTK models allow for incorporation of in vitro data and in silico predictions to derive human equivalent doses [28]. Consideration of variability of these data allows for extrapolation of a range of in vivo equivalent doses across individuals rather than a single value, which shall more accurately reflect the distribution of population responses that occur from exposure to a chemical [29].

This review will describe major drivers that contribute to TK/TD variability and provide background on the structure and evolution of TK and PBTK models, designed to address a varied range of exposure scenarios across a wide range of data inputs. This will be followed by a state of the science update describing current and emerging modeling strategies and emerging experimental and modeling strategies that incorporate population and life-stage variability, including the increasing application of IVIVE with PBTK models in pharmaceutical and chemical safety research. The review will conclude with case study examples demonstrating novel applications of PBTK modeling that demonstrate the power and potential of these models to evaluate variability.

## Drivers of human TK variability

In order to provide context for incorporation of variability into PBTK modeling, it is necessary to understand the key drivers of human TK variability. Factors that contribute to population TK variability can be divided across four different groups: physiological, ontogenetic, genetic, and exposomic. Table 1 provides a summary of these factors and considerations regarding their impacts on variability.

**Table 1:** Contributors to TK variability

Contributors to variability	Effect window	Extent of effect	Frequency
Physiologic (e.g., tissue weights, blood flow rates)	All life-stages: early and late—greatest; disease	Moderate	All populations and life-stages
Genetic (functional differences in enzymes, transporters)	All life-stages	Depends on polymorphism, functional effects	0–10% of population
Ontogenetic (differing abundances in enzymes, transporters)	Early life-stages	Can be significant	All individuals within relevant life-stages
Exposomic (e.g., co-exposures, lifestyle, microbiome)	Throughout life	Dependent on cause; (chemical interactions; bioavailability)	Active research area

### Physiological factors

Differences in the underlying physiology involved in ADME can contribute to variations in TK. Although variability does indeed exist within and between individuals within the same life-stage, notable physiological differences are more pronounced when comparing adults (e.g., 20–50 years old) to children or elderly (e.g., > 60 years old), or groups with differing health conditions/status (e.g., heathy subject vs. one with liver disease, non-pregnancy vs. pregnancy) [30–33]. The impact of physiological differences is perhaps best conveyed through comparisons between young and elderly adult populations (e.g., 25-year-old vs. 65-years-old). The elderly generally show reduced hepatic metabolism and renal clearance due to a variety of age-related physiological changes, such as reduced liver blood flow, a reduction in hepatic cytochrome P450 (CYP) half-life, and a reduction in muscle mass and water content [30]. Both the fat-to-muscle ratio and water content can change the volume of distribution (V_d_), the theoretical body volume in which a drug is distributed to reach the same concentration observed in the plasma, e.g., an increase in the former, or loss of the latter would increase the V_d_ for lipophilic compounds and decrease the V_d_ for hydrophilic compounds. Moreover, there is a reduced ability in the elderly to counter environmental exposures and stressors, as evidenced by an accumulation of lipofuscin, which has been linked to a drop in the ability to offset oxidative stress, e.g., a decline in brain antioxidant levels [34]. In line with this, brains in the elderly show an increased sensitivity to neurotoxicants, which may in part be caused by reduced activity of acetylcholinesterase, the enzyme that breaks down the neurotransmitter acetylcholine, and reduced functional reserves of the aging brain [30]. In addition, liver and kidney disease are more common in the elderly, further altering metabolism and clearance as discussed below [30].

Alternatively, infants and children are characterized by differing physiologic composition as their bodies evolve and develop into adults [35]. Infants up to three months of age have an elevated water: lipid ratio, generally resulting in an increase in the half-life and V_d_ of hydrophilic chemicals [31, 36]. Children up to six years of age have a larger brain to body weight ratio and greater blood flow to the central nervous system, thereby potentially increasing the distribution and half-life of a chemical in the brain [31, 37–39]. Other physiological differences include tissue volumes and blood flow, and immaturity of respiratory, renal, and hepatic systems in children [40]. Although more of an exposure factor, children also generally have increased inhalation and food intake in proportion to body weight. This is of particular concern due to children’s increased exposure to soil and house dust, which can be contaminated with toxicants [41]. There is also evidence for differences in the absorption of chemicals such as an increase in gastrointestinal absorption of lead, inorganic mercury, and other metals in children [31].

Apart from children and the elderly, TK differences are observed in numerous disease populations. Perhaps the most notable of these is liver cirrhosis. Numerous liver diseases exist, with cirrhosis being the most common, and itself having numerous etiologies, which may differentially influence PK [42]. In general, cirrhosis results in impaired hepatocyte function, changes in blood flow, and a reduction in plasma proteins, which subsequently result in changes in drug clearance due to altered liver size, CYP expression, plasma protein binding, and hepatic blood flow [43, 44]. Changes in glomerular filtration rate are also frequently seen [43]. Numerous studies have shown a reduction in enzyme and transporter expression in cirrhosis that correlates with disease severity; these generally result in a reduction in clearance and increase in the area under the curve (AUC) [43, 44]. Non-alcoholic fatty liver disease is the primary contributor to cirrhosis, even becoming more common in pediatric populations, and has its own set of symptoms, including changes in gastrointestinal pH [42]. Many symptoms of non-alcoholic fatty liver disease can be linked to comorbidities, such as obesity, which is the likely source of the reduction in cardiac output and CYP2E1 activity and expression seen in non-alcoholic fatty liver disease [42].

Indeed, obesity, which now affects 42% of adults in the U.S., results in a number of physiological and biological changes that influence TK [45–47]. The elevated body mass index of obesity increases cardiac output and liver and kidney weight and blood flow, while adipose blood flow is decreased [45, 47]. These physiological changes result in an increase in the V_d_ of lipophilic compounds and subsequently their half-life [46, 47]. Apart from increases in clearance by CYP2E1, there is also a reduction in CYP3A4 expression [42, 47]. Changes in plasma protein levels are also seen [47].

Another disease that results in significant alterations in PK, including in clearance, transporter function, and V_d_, is chronic kidney disease [48, 49]. Kidney disease results both in a reduction in glomerular filtration rate as well as in active tubular secretions [50]. Plasma protein binding is also altered, causing an increase in uremic solutes and their subsequent accumulation in blood and tissues, which has been associated with a reduction in hepatic metabolism and both hepatic and renal transport [50]. PK is affected not only for renally eliminated drugs, but also for non-renally cleared drugs, particularly those cleared through the CYP2D6 pathway [49]. Each of these factors tend to increase with disease severity [48].

Recent work shows that one critical driver behind the changes in PK that result from disease, particularly chronic inflammatory diseases, seems to be inflammation [44]. For instance, the reduction in enzyme and transporter expression seen in liver cirrhosis has been linked to the upregulation of inflammatory cytokines [44]. An elevation in inflammatory markers and adipokines is also seen in obesity [45]. For additional perspective on the impact of inflammation on TK, we refer the reader to a recent review [51].

### Genetic factors

Genetics can play a major role in interindividual variability throughout the lifespan, depending on the functional consequences of the genetic variant and the compound [52]. Polymorphisms have been shown for nearly all the CYPs, resulting in functional variability, particularly for CYP2D6 and CYP2C19 activities, leading to differences in metabolism and subsequently in chemical half-lives [53]. CYP2D6 is commonly cited as an example of the role of genetic variability on interindividual variation due to its major role in metabolism of pharmaceutical compounds and wide distribution in patient responses. There are over 133 CYP2D6 variants, leading to 60–100-fold variation in CYP2D6 activity [10]. Varying phenotypes resulting from common alleles can be grouped as poor metabolizers—those that show an increased and delayed AUC, or extensive metabolizers and ultrarapid metabolizers—those that show a lower, shorter, AUC (Fig. 1) [54]. Poor metabolizers make up 1–8% of the population, intermediate metabolizers 0.4–11%, normal 67–90%, and ultrarapid 1–21%, depending on ethnicity [55, 56]. Other CYPs that have shown variability with clinical relevance include CYP2C9, the primary enzyme in warfarin metabolism, and CYP2C19, the major enzyme in omeprazole metabolism. Dosing for warfarin is commonly determined based on CYP2C9 genotype of the patients [57]. Phase II enzymes can also exhibit phenotypic variation. Of the Phase II uridine 5'-diphospho-glucuronosyltransferases (UGTs), UGTs 1A1, 1A7, 2B15, and 1B17 are the enzymes most likely to show functional consequences [52]. Sulfotransferase 1A1 is also suggested to have phenotypic variations. Further details on six such polymorphic xenobiotic metabolizing enzymes (i.e., CYP2D6, CYP2E1, aldehyde dehydrogenase 2 (ALDH2), paraoxonase 1 (PON1), glutathione transferases (GSTs), and N-acetyltransferases (NATs)) can be found elsewhere [12]. Polymorphisms in transporters may also contribute to TK variability, one example of which has been reported for organic cation transporter 2 (OCT2) [58].

The frequency of certain polymorphisms varies by ethnicity, which contributes to demographic differences in metabolism. Allelic frequencies for many metabolizing enzymes have been estimated, particularly for the groups most commonly studied, such as Caucasians and Japanese [59, 60]. Variation is not solely due to polymorphisms in the coding region of the enzyme. The phenotypic variability may also arise from variants in noncoding regions such as the promoter, microRNAs that regulate expression, and other transcriptional regulators [10]. While many polymorphisms have been identified, not all of these have been characterized, limiting the ability to draw conclusions on functional consequences of these polymorphisms. It should also be noted that polymorphisms may lead to heightened or poor metabolism, which may or may not lead to an adverse outcome; so although TK variability may be noted, whether it results in TD differences needs to be determined [12].

### Ontogenetic factors

Ontogenetics, also known as developmental pharmacology, refers to the study of how compounds are differentially processed and metabolized in the body throughout the course of development. It plays a major role in exposure response variability in children, which particularly involves enzyme and transporter ontogenies [40, 61]. Most metabolic enzymes are present at very low levels, if at all, during fetal development [31, 62]. CYP3A7 is an exception, with its highest expression during the first trimester, staying stable throughout gestation, until its expression levels fall after birth, when CYP3A4 begins to predominate among the CYP3As. Some enzymes, such as CYPs 2C9, 2D6, 2E1, and 3A4, and UGTs show a rapid increase in expression after birth, though this is highly variable [62, 63]. Other enzymes are slower to express, such as CYP1A2, which does not reach adult levels until approximately one year of age [31, 64]. Despite a lack of certain enzymes, there may be metabolic compensation for some compounds due to substrate overlap. For instance, CYP3A7 shares many substrates with CYP3A4, and sulfotransferases share several substrates with UGTs, providing alternate routes for these to be conjugated and eliminated [52]. Intra- and inter-study comparisons show that there are windows of biological hypervariability in enzyme development, adding yet another layer of complexity to neonatal metabolic function [65]. Although life-stage-specific abundances of CYPs and UGTs readily involved in drug metabolism are well-characterized, several enzymes involved in non-drug chemical metabolism have only limited data available, leading to greater uncertainties in such evaluations. As the field matures more data are being collected [66–68]; indeed, more experimental data are needed to provide greater confidence in their application, in modeling or in development of quantitative structure–property relationship prediction tools.

Evaluation of transporter abundance and developmental changes will also be important in characterizing TK variability. As membrane-bound proteins present in numerous tissues (e.g., kidney, liver, brain, placenta), transporters actively uptake or efflux endogenous and exogenous compounds, thus impacting chemical distribution in the body [69, 70]. With the emergence of targeted proteomics as a robust experimental tool for membrane protein abundance, tissue-specific, ontogenetic evaluations have been a highly active research area in recent years [16, 71]. It is worth noting that levels of P-glycoprotein (P-gp), an efflux transporter present in several metabolic tissues, such as brain, placenta and mammary tissue, have consistently shown lower levels in preterm and newborn samples compared to older children and adults [72]. An investment in understanding age-dependent abundances of placental and mammary transporters will yield critical information that could be directly applied in maternal–fetal PBPK models to evaluate fetal drug or chemical exposure [73]. Recent efforts that compile documented interactions between environmental pollutants and transporters have noted a range of activities, spanning from substrates for efflux transporters (endosulfan, methoxychlor: P-gp); uptake transporters (2,4-D, Aflatoxin B_1_: organic anion transporter 3). PBTK models will be critical in broadening our understanding of transporter involvement in TK variability.

### Exposomic factors

Exposomics aims to comprehensively assess all types of exposures and/or stressors that an individual may encounter over their lifetime [74]. Exposomic factors cover a huge range, from lifestyle-related to environmental and geographic factors, and have the potential to modulate both TK and TD variability. Alcohol and tobacco consumption, diet-related factors (e.g., nutritional status, cruciferous vegetable intake), obesity, and contraceptive use, all impact expression of a range of Phase I and II metabolic enzymes [52]. Xenobiotic co-exposure through drug use, particularly polypharmacy, can often impact metabolism, with drug-drug interaction a major research area for the pharmaceutical industry [75]. This is particularly relevant for the elderly, who are often taking many medications at once. Apart from drugs, other xenobiotics may impact metabolic rates, such as cadmium and lead exposure, or exposure to environmental carcinogens more generally [76]. It is of particular note that xenobiotic exposures may have critical windows of susceptibility, most of which occur during embryonic development or the first few years of life [77]. In utero chemical exposures have been shown to cause gene expression changes in the fetus, which could lead to changes in enzyme expression. For children, it is important to also consider that they have more hand-to-mouth activities and floor contact.

Consideration of the influence of gastrointestinal microbiota on metabolism is an emerging exposomic factor of relevance for both TK and TD variability [19]. Although unlikely to be important for xenobiotics rapidly absorbed in the upper small intestine, it may be a factor for low-solubility, low-permeability compounds and those subject to biliary excretion [18, 78]. Interindividual host microbial genome differences have been attributed to differences in gut microbiome composition and drug metabolism [79, 80] and as such are likely to elicit variability in microbial-compound metabolism. Moreover, many environmental pollutants have been shown to be subject to the reductive and hydrolytic microbial enzymes such as azoreductases, nitroreductases, and β-glucuronidases, including nitrotoluenes, pesticides, polychlorinated biphenyls, and azo dyes [81]. Additionally, many of these chemicals can affect or inhibit microbiome composition as demonstrated in animal studies, including chlorpyrifos, bisphenol A, and traffic-related air pollution [82–84]. The interplay between these interactions and their implications for human health assessment are quite complex, still requiring significant elaboration across multiple experimental models. More analysis and insights can be found in two excellent reviews on this topic [18, 19].

Until recently, an often overlooked exposomic factor is societal, which encompasses a myriad of factors including dietary, social, and environmental stressors. Consideration of geographic factors that can impact xenobiotic exposure, such as the toxic substance profile of the area, housing quality (e.g., asbestos and paint), the types of heating and cooking, pest control methods, urbanization (e.g. traffic-related air pollution), and climate (e.g., exposure to diseases and the types of outdoor play areas) is important [85]. Moreover, these factors are often related to environmental justice, with certain populations experiencing a greater burden than others, e.g., non-white people living in areas with increased pollution and toxicant exposure propagated from financial service discrimination (i.e., red-lining), with simultaneously elevated stress due to housing and economic pressures [85–87]. Indeed, a consensus recommendation following a 2020 National Academies of Science Engineering and Medicine workshop focused on strategies to integrate aging and environmental health research called for the implementation of a compound exposome approach to ensure adequate consideration of unique social and neighborhood factors [88].

### Additional considerations

Apart from the discussed contributors to variability, it is likely apparent that delineations between such factors are not always clear-cut. Some population characteristics, such as sex, disease, and ethnicity, contribute to TK variability in overlapping yet distinct ways; consideration of population inputs regarding physiology and genetics for instance may require a nuanced approach. Another potential confounder in this field is the bias of available data, as the majority of studies are based on pharmaceutical compounds and healthy Caucasian individuals. Relevance to environmental pollutants possessing disparate physicochemical properties and other subpopulations is unclear without further evaluation. On the other hand, not all factors that may contribute to variability will have an impact on TK or TD leading to an adverse effect. For instance, if blood flow is the limiting factor to metabolism, variability in metabolic rate or enzyme levels may not impact steady-state levels [12]. Variability in metabolism and whether it is ultimately linked to an adverse outcome depends on whether such metabolism leads to clearance from the system, or formation of a reactive, toxic metabolite [9]. As such, problem formulation and case-by-case considerations are important to ensure TK and TD variability are appropriately considered and evaluated for decision-making.

## PBTK modeling and incorporation of variability

### PBTK modeling

PBTK methods can range from noncompartmental—where xenobiotic blood concentrations per unit time measurements are used to inform estimates—to compartmental methods that can represent varying degrees of complexity. The concept of multi-compartmental PK models for the simulation of PK data was introduced as early as 1937 (Teorell), but widespread use has only occurred recently largely due to initial limitations of mathematical complexity and presumed data requirements [89]. The advent of reliable tools to predict required inputs (e.g., V_d_) using physicochemical properties has facilitated increased usage [89–91]. Initial problem formulation is required to ensure all relevant scenarios for all relevant ADME characteristics are considered: for instance, which exposure routes are relevant (e.g., dermal, inhalation, ingestion) will in turn dictate which routes of absorption need to be addressed and how bioavailability (i.e., amount of chemical reaching the bloodstream) is to be calculated [2].

**Fig. 2:**
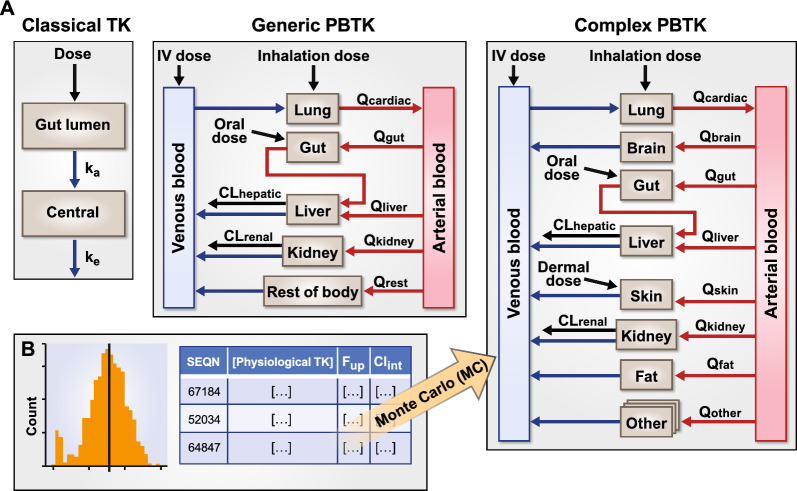
Evolution of PK Models. **A**. General structure of Classical TK, Generic PBTK, and Complex PBTK models. **B**. Monte Carlo (MC) simulations can be used to incorporate interindividual variability. k_a_: rate of absorption; k_e_: rate of elimination; CL: clearance; Q: blood flow

**Fig. 3:**
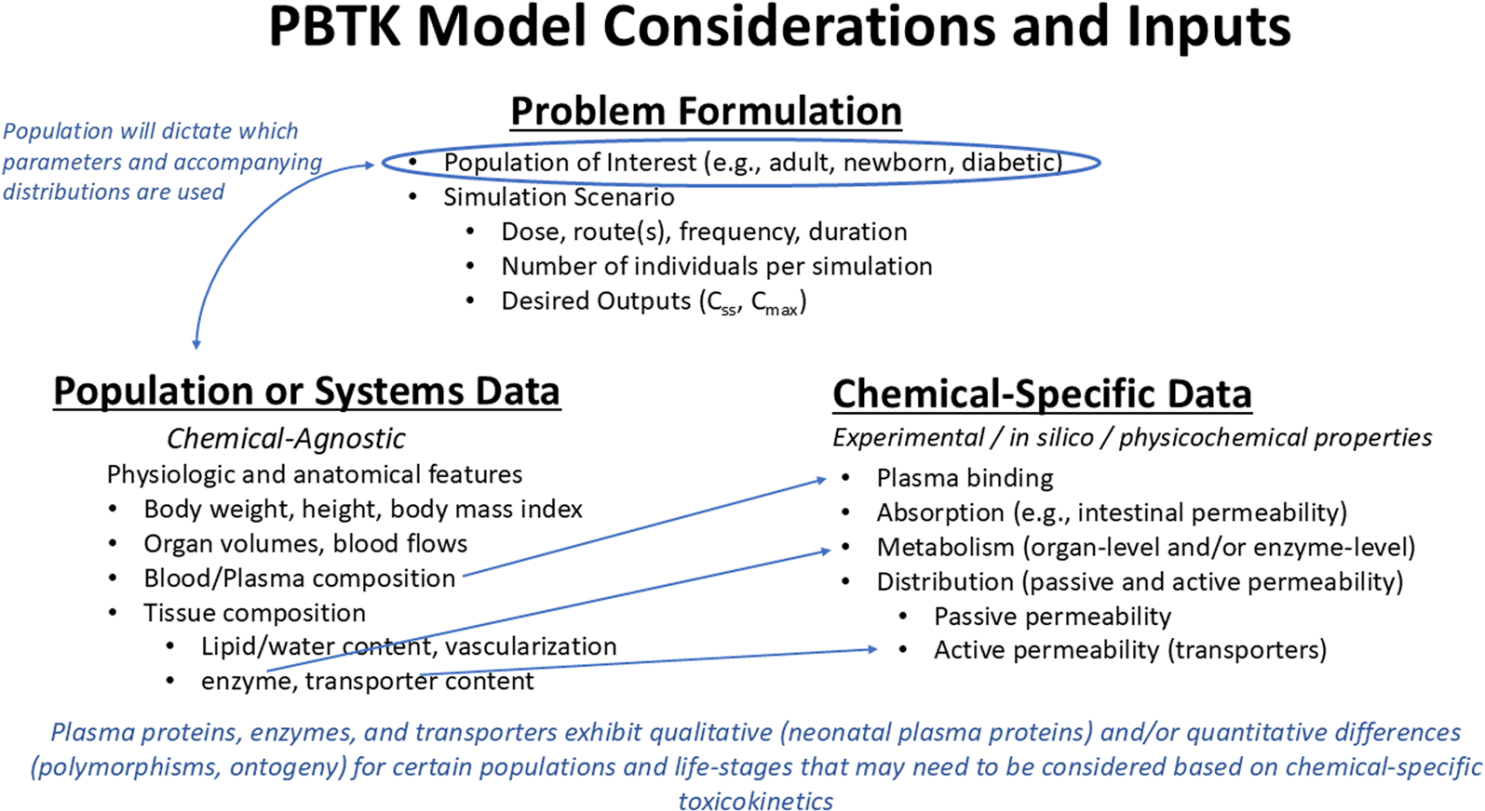
PBTK model input streams and considerations

Compartmental models can range from more simple forms that consider little more than a central plasma compartment connected to peripheral compartments, with the application of rate constants to estimate PK; to more complex models that incorporate multiple tissue types and accompanying biology and physiology (Fig. 2) [92]. In more complex PBTK models designed to evaluate population variability, input parameters are described as either system-level (i.e., population-specific) or chemical-specific, wherein either TK inputs for a particular chemical and/or the accompanying physicochemical properties are considered to derive the needed input parameters (Fig. 3). If in vitro or in silico estimates are employed, in vitro to in vivo scaling may be required. These approaches of using in vitro or in silico measurement to estimate parameter values for populating PBTK models are referred as a bottom-up approach [93]. To incorporate variability in such approaches, information on the variability (e.g., probability distributions of a parameter for the population being modeled) can be incorporated during model simulation, as captured in Fig. 2.

### PBTK modeling and variability simulation

Incorporation of variability in PBTK models can be performed to capture both physiological and biochemical variability to simulate TK in a population of individuals rather than an average subject. Equations describing distributions of system parameters for a model are derived from distributions of data based on real populations or patients. PBTK models can then be used to estimate internal concentrations from external exposures for these virtual individuals, which can arise from any population or life-stage of interest for which such data are available. The proprietary software Simcyp, a modeling and simulation software supported by a consortium of pharmaceutical companies with a mutual need for modeling and simulation [55], has a varied range of preparameterized population libraries that encompass human gestational, pediatric, and elderly life-stages and several ethnic and disease populations, as well as a few preclinical species [43, 94, 95]. Of the open-source tools designed specifically for chemical PBTK modeling, PopGen possesses a virtual human population generator that can be used to predict anatomical, physiological, and Phase I metabolic variation in healthy humans using human biomonitoring data [96]. Another platform designed specifically for NAM chemical risk evaluations developed at the United States Environmental Protection Agency (EPA) is the httk R package, which can make predictions of TK profiles for multiple chemicals in various species [97]. The httk package includes different types of TK models with varied complexity that allow the incorporation of in vitro TK data, physicochemical properties, and species-specific physiological data. With each type of TK model, the plasma or tissue concentration–time profile can be generated using forward dosimetry for individuals or populations. In the simplest model of the httk package, only intrinsic clearance rates (Cl_int_) and fraction unbound in plasma (f_up_) values are needed to run the model.

### Virtual population simulations to capture physiologic variability

Both Monte Carlo (MC) simulation and Bayesian approaches can be used to incorporate population variability in PBTK modeling by generating and simulating virtual populations. MC, an approach that can be correlated to ensure variability is constrained to biologically feasible limits, is the preferred method for incorporating population variability [94, 96–98]. One example of MC application is in the httk package HTTK-POP, which incorporates interindividual variability for ten U.S. demographic groups based on National Health and Nutrition Examination Survey (NHANES) biomonitoring data [99]. To develop the representative populations, MC sampling of physiological parameters for the ten demographic groups from the NHANES cohort was performed, and then used to simulate interindividual physiological variability for each group, as well as experimental variability in f_up_ and Cl_int_. As isozyme-specific information was not available, five percent of the population was assumed to be poor metabolizers. Exposure information was also considered for each of the demographic groups, showing the elderly population to be at the greatest risk, due to reduced clearance.

With incorporation of measures of human TK, such as Cl_int_ and f_up_, the distributions of doses that could elicit bioactivity can be predicted, providing a measure of interindividual variability. Interindividual TK and physiological variability have been incorporated in an IVIVE-PBTK approach using Simcyp [29]. The Simcyp PBTK model offers advantages over other PBTK models for assessing interindividual variability in TK and TD, as it provides a range of subpopulation and life-stage libraries that include information on variation in isozyme expression as well as physiological characteristics [29, 100]. Information on isozyme expression allows for consideration of both genetic and ontogenetic contributors to TK and TD variability, providing more accurate estimates of Cl_int_, particularly for children under 5 years of age [94, 100, 101]. Wetmore et al. generated isozyme-specific clearance rates on nine chemicals across 13 different Phase I and II enzymes and incorporated this data into Simcyp, performing PBTK modeling for a range of sensitive groups to identify the degree of variability in steady-state concentration (C_ss_) concentrations [29]. This bottom-up approach utilizing Cl_int_ values at the level of individual isozymes allows great flexibility in revising system parameters, such as for new demographic, genetic and physiological data to make assessments of interindividual variability in TK parameters. Several other studies have incorporated TK variability using similar approaches [102, 103]. A study on methyleugenol used both individual human liver fractions and specific isozymes to estimate clearance rates and combined this in vitro data with literature-derived interindividual variability in a PBTK model with a MC simulation approach [102]. A study by the same group incorporated a developmental endpoint for calculating a phenol chemical-specific adjustment factor (CSAF), making for a semi PBTK-PD approach. Interindividual variability in oral absorption and UGT expression were incorporated into the PBTK model using MC simulation [103].

PBTK modeling can also help to elucidate the key drivers underlying the manifestations of population variability in PK seen in disease, as well as optimize dosing in disease populations [43, 44, 46, 49]. Indeed, the European Medicines Agency supports the use of PBTK models for clinical study design and a number of commercial and open-source PBTK modeling platforms include disease populations in their platforms [43, 44, 104, 105]. These populations can be continuously refined based on additional data.

Moreover, sensitivity analysis can be performed to identify the major drivers of interindividual variability. Al-Subeihi et al. [102] found bioactivation by CYP1A2, epoxidation by CYP2B6, and the apparent kinetic constants for oxidation and sulfation to be the greatest contributors to metabolic variation between individuals. Grzegorzewski et al. [106] explored the contribution of physiological parameters on CYP2D6 metabolic activity, showing these to have little impact and contribute similarly, independent of CYP2D6 activity. In a PBTK model of bromodichloromethane in pediatric populations, CYP2E1 was found to be the greatest contributor to variability in pharmacokinetics [107]. Such sensitivity analyses are useful in focusing efforts to better understand population variability.

## IVIVE

IVIVE, a process using in vitro data to predict in vivo phenomena, is a critical translational step in the conversion of NAMs into metrics that are useful in risk assessment. IVIVE is used both to scale in vitro measures of TK parameters to the corresponding in vivo parameter, or to convert an in vitro concentration, typically related to assay bioactivity, to an in vivo effect dose, which can be compared against in vivo data or exposure [28].

IVIVE to support TK modeling requires assay- and parameter-specific considerations are met to ensure quality data are generated and appropriate scaling is achieved. Many of the assays designed to evaluate key parameters such as plasma protein binding and hepatocyte clearance have undergone rigorous evaluation as they were developed for use in the pharmaceutical industry, with more recent evaluations conducted to support guidance provided by the Organisation for Economic Cooperation and Development (OECD) [108–110]. Good in vitro method practices ensure that approved methodologies are followed, in conjunction with the associated negative and positive controls and any reference compounds to ensure assay reproducibility is maintained. Additional consideration is given to the use of extrapolation factors that may be required in instances where variances across in vitro systems (e.g., hepatocytes vs. microsomes) or assay conditions (differing but acceptable pH conditions) require certain adjustments for normalization. Finally, scaling factors required to scale up to represent organ-level clearance may vary, depending on the population or species of interest.

### Incorporating TK and high-throughput screening data for equivalent dose estimation

IVIVE can also translate a bioactivity concentration from an in vitro assay (which can include TK/TD variability) into human-relevant doses. In this type of IVIVE application, reverse dosimetry is performed by incorporating chemical TK, which is typically realized by using a PBTK model, and to calculate an in vivo equivalent dose that could lead to a plasma or tissue concentration equal to the in vitro bioactivity concentration. The typical method to calculate the in vivo equivalent dose is described below:

$${\text{Administered}}\; {\text{equivalent}}\; {\text{dose}}\; \left( {\frac{{\frac{{{\text{mg}}}}{{{\text{kg}}}}}}{{\text{d}}}} \right) = \left( {\frac{{{\text{1mg/kg/d}}}}{{{\text{C}_{\text{ss}}}\left( {\mu {\text{M}}} \right)}}} \right)*{\text{in vitro}} \;{\text{bioactive}} \;{\text{concentration}}\; \left( {\mu {\text{M}}} \right)$$

Alternate methods to calculate points of departure for risk assessment are available as well, such as based on dose–response curves [111]. A key assumption to operationalize this IVIVE approach is that human plasma concentrations are equivalent to in vitro assay media concentrations. While nominal assay chemical concentrations are typically assumed to be the concentration available to elicit an effect, differential partitioning of chemicals between plastic, cells and media may affect the accuracy of such nominal concentration-based estimates, leading to a potential over/underestimation of bioactivity [112, 113]. Current practice uses nominal concentrations due to limited empirical data to evaluate available models; however, ongoing efforts to refine existing models, curate assay-specific details, and generate evaluation data may yield a path forward to incorporate distribution characteristics in future potency estimations [114–116].

**Fig. 4:**
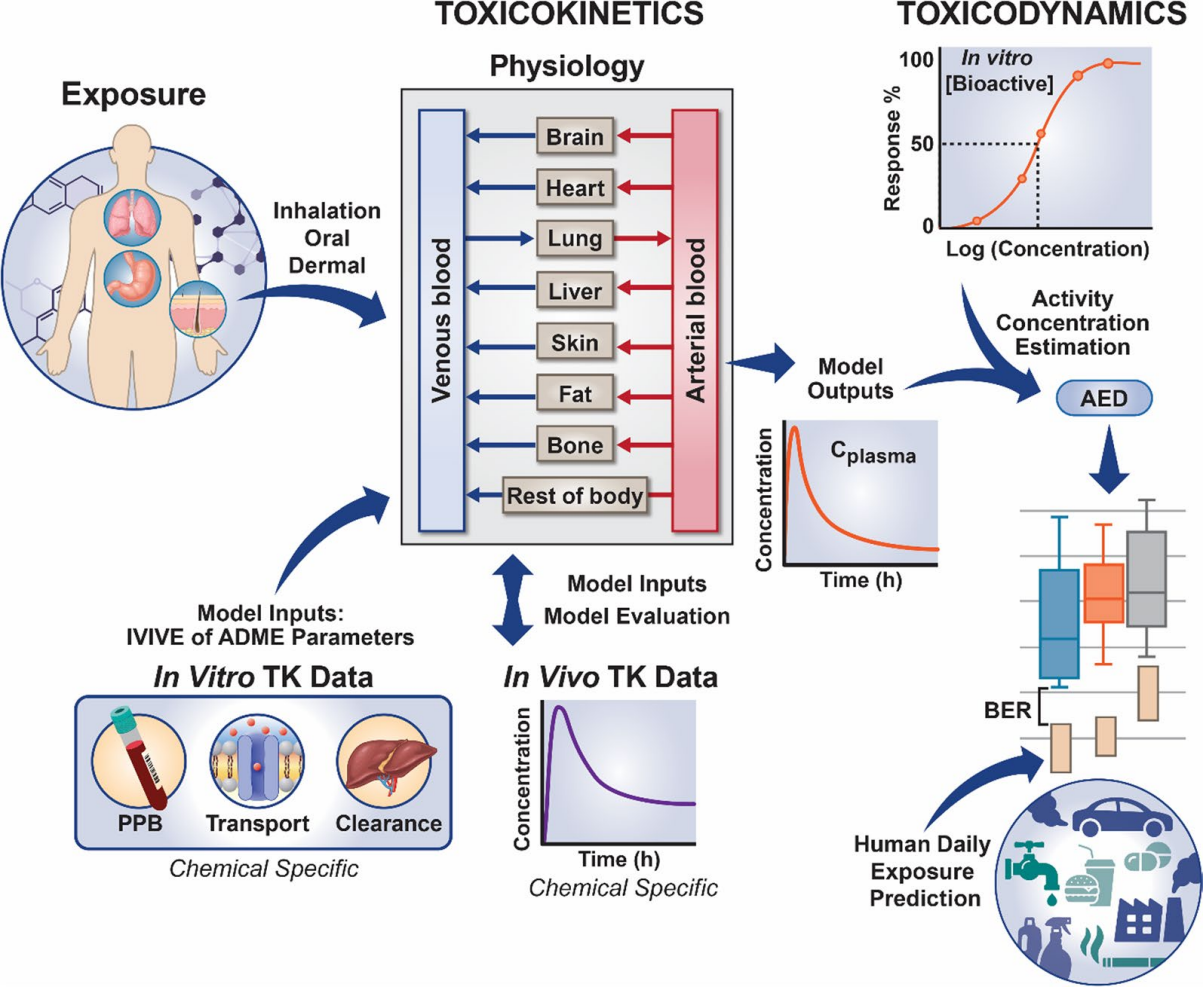
General PBTK-IVIVE workflow. Various exposure routes can be simulated. In vitro TK data is generated and used to inform PBTK model through IVIVE of ADME parameters. In vivo TK data (e.g., plasma protein binding (PPB), transport, hepatic clearance) can also be used to inform the PBTK model. Estimated internal concentrations (e.g. C_plasma_) are combined with in vitro toxicodynamic points of departure to derive an administered equivalent dose (AED), representing an external exposure that could elicit a similar bioactivity in vivo. These AEDs are compared against human exposure estimates to derive bioactivity exposure ratios (BERs), an ad hoc margin of exposure

The general PBTK-IVIVE workflow is outlined in Fig. 4. IVIVE for TK variability is perhaps best demonstrated in Wetmore et al. in which measured chemical TK parameters—hepatic clearance and f_up_—were incorporated into an IVIVE-PBTK approach [117]. In addition, in vitro bioactivity data were incorporated to derive the oral equivalent dose, or administered equivalent dose (AED), that would produce a C_ss_ that might elicit bioactivity.

### Exposure risk evaluation using bioactivity data: bioactivity exposure ratios (BERs)

Using IVIVE, in vitro effective concentrations can be used to generate AEDs which can then be compared against exposure estimates to derive a BER, a margin of exposure metric, to inform chemical prioritizations for risk assessment [118]. The lower the ratio, the higher the potential for the exposure to elicit bioactivity.

In vitro bioactivity data for IVIVE can be obtained from large scale screening studies performed across numerous chemicals and covering a range of endpoints. Two such major data resources from the U.S. are the Tox21 and ToxCast programs. Tox21/ToxCast is a collaborative effort between the National Institutes of Health (NIH), FDA, and EPA to develop and implement in vitro high-throughput assays to screen thousands of chemicals across hundreds of bioactivity or toxicity endpoints [11, 118]. Chemical potency from these assays is commonly summarized by an activity concentration exerting 50% of maximum response (AC50) or lowest effective concentration value [119], although other metrics can be used to define in vitro points of departure. It is worth noting that while the Tox21 and ToxCast assays provide a valuable resource for obtaining in vitro bioactivity data, due to their high-throughput nature, potency values derived from these assays should not be taken at face value without a thorough review of data quality underlying concentration-response curves. Bioactive concentrations should always be confirmed, and potential flags such as borderline activity, curve overvitting, and background noise should be carefully assessed. The subsequent chapter will highlight some of the NAMs employed in these IVIVE and risk assessment approaches.

## NAM applications of IVIVE and modeling to inform chemical risk evaluations

### NAMs incorporating metabolism and physiology to quantitate life-stage TK variability

Some measures of human TK variability are well-characterized as they are readily measured or obtainable—these are primarily physiological parameters, such as blood flows and tissue volumes [120]. In contrast, fewer data are available for the effect of genetic and ontogenetic factors, such as clearance rates, and far less is known regarding exposomic factors. NAMs here hold a particular advantage in that they can be performed using human-specific biology and in a high-throughput manner, allowing for an increased sample size, which is necessary when evaluating interindividual variability. Such NAMs include in vitro assays for both TK and TD variability, ranging from subcellular measures of metabolism and transport to cellular and tissue-level endpoints, and in silico prediction tools [29, 121].

In vitro assays that can measure human TK variability exist for most tissue types, including liver, lung, and skin [24, 122]. Wetmore et al. developed a screening approach to characterize TK variability across different populations and life-stages by measuring isozyme-specific clearance rates [29]. In this initial approach, a set of nine chemicals was screened across 13 isozymes. Another major contributor to TK variability is transport of chemicals. Several in vitro approaches are available to measure transporter activity. One common technique is to overexpress certain transporters in cell lines, such as HEK293 or Caco-2 cells, and then measure cellular uptake of compounds [58]. In addition, inhibitors can be used to measure the contribution of individual transporters [123]. With the incorporation of known variation in transporter expression between populations and life-stages, these measures can help to estimate variability in transport process and subsequent C_ss_ using PBTK modeling.

### NAMs for exposure prediction

While emphasis One major resource for deriving human exposure estimates in the U.S. is the NHANES study from the Centers for Disease Control, which measures exposure biomarkers—primarily metabolites—from a diverse array of individuals across the U.S. in both blood and urine [124]. To expand use of human exposure data from NHANES and incorporate the National Research Council (NRC) request for predictive tools to be used for safety assessment, ExpoCast was developed by the EPA as a high-throughput exposure prediction model [125]. In addition to chemicals measured in NHANES, ExpoCast provides exposure predictions for thousands of additional chemicals, covering nine life-stages and demographic groups.

### TD variability

While emphasis is often placed on TK variability, TD variability is another driver of population variability [52]. One of the first NAMs developed to assess human TD variability used a set of 146 lymphoblastoid cell lines that were characterized through the 1000 genomes project [11]. This population genomics approach has particularly helped to characterize genetic contributors to human population variability. It has also been used to characterize TD variability under complex scenarios, such as in response to chemical mixtures and identifying a chemical’s mode of action. TD variability can also be assessed in in vitro studies using donated human samples obtained through clinical trials. These NAMs, such as those from Genoskin and the Institute for In Vitro Sciences, use patient samples from a diverse pool of donors covering a range of ethnicities and ages, which can be used to assess interindividual variability for endpoints such as efficacy and immune responses [122, 126].

An exciting novel avenue to address interindividual variability has appeared with the development of methods to culture human induced pluripotent stem cells (hiPSCs). hiPSCs can be generated from somatic cells of individuals with different genetic backgrounds, which can be used to represent the genetic diversity of the population. Burnett et al. developed a model to measure TD population variability in cardiomyocytes derived from hiPSCs that shows strong reproducibility, sensitivity, and specificity [121]. Using hiPSCs from 43 individuals, a size similar to that used in human clinical trials, Burnett et al. screened 134 chemicals for functional and viability endpoints. They found interindividual variability to be the primary contributor to total variability, with little contribution from sex and ancestry. Using such an approach, they were able to characterize interindividual variability in cardiomyocyte responses and look at specific genetic contributors to human TD variability. Due to their human-relevance, usability in biochemical, genetic, and genomic approaches, and their capacity for assessing endpoints and mechanisms, NAMs using hiPSCs are being developed for a range of other tissues and endpoints [127, 128].

TD can be linked to PBTK models to create a PBTK-TD model [129]. This model allows for tying the concentration at the site of action to the downstream effect of the compound and can help to provide mechanistic information underlying this effect. Further details on such PBTK-TD models can be found elsewhere [130, 131].

## Consideration of population variability in regulatory decision-making

Incorporating IVIVE and modeling approaches to inform population variability has a role in several arenas, including to support pediatric drug development and dose selection in the pharmaceutical industry [132], and informing setting testing priorities and evaluations of population variability in the toxicology arena [27, 118]. In the pharmaceutical industry, PBTK modeling and simulation (i.e., model-informed drug development) can help determine clinical study needs, study design including dosimetry, and the need for population-specific product label warnings [133]. In the toxicology arena, the release of two NRC reports—“Toxicity Testing in the 21st Century”, advocating for the use of what were to become NAMs to inform toxicity assessment [134], and “Science Decisions: Advancing Risk Assessment,” advocating for the need to include population variability in toxicity testing [135], spawned numerous efforts utilizing IVIVE and PBTK models to evaluate population variability [29, 117, 118, 136–138].

### UFs and CSAFs

Population variability has traditionally been accounted for with the use of uncertainty factors (UFs) in risk assessment, with the default UF for human safety assessment using animal data as 100X, consisting of a 10X factor for interspecies variation and a 10X factor for interindividual variability [139]. The 10X for interspecies can further be broken down into 4X for TK and 2.5X for TD, and the 10X for interindividual variability into 3.2X for both TK and TD, allowing for adjustment of these different types of uncertainties when additional information is available [140, 141]. These factors and subfactors have been adopted by organizations worldwide, including the World Health Organization (WHO)/International Programme for Chemical Safety (IPCS), European Food Safety Authority, Health Canada and the EPA [20, 27, 142].

In those instances where quantitative data on TK and TD are available, most commonly in a higher tier risk assessment, there is an opportunity to depart from the default UFs in favor of CSAFs. Guidance advising on CSAF derivation was published in 2005 by WHO/IPCS, an outcome of an international harmonization project [142]. In essence, availability of data-derived factors that allow grouping due to 1) non-mode of action information (e.g. allometric scaling, clearance-related) or 2) mode of action-related that incorporates TK and/or TD data allows for biologically-based adjustment factors that reduce uncertainty estimations, allowing for departure from the default UF.

Some investigative (i.e., non-regulatory) evaluations using modeling have shown that a UF of 10X may be insufficient for certain populations and life-stages, including children and the elderly, and for chemicals metabolized by polymorphic enzymes [143, 144]. Strikwold et al. [103] provided an example of a CSAF approach for phenol in which a CSAF of only two was calculated based on the degree of interindividual variability in the maximal plasma concentrations. Wetmore et al. found the degree of variability, indicated by the human TK adjustment factor (HK_AF_), to vary from 1.3 to 13.1, with the pediatric life-stage typically showing the highest internal dose (C_ss_) [118]. In an effort that estimated TK and TD variability for complex mixtures, Abdo et al. found that the default UF aligned well with the TD variability of the two mixtures tested, although whether this result holds for other mixtures is unknown [145]. Uncertainty estimation relevant for the specific problem under study is also an important consideration that requires further elaboration [146].

A recent state of the science update [63] reviewed regulatory and investigative CSAF development and consideration by regulatory bodies to provide next steps to foster future adoption of CSAFs. Preferably, CSAF development based on PBTK models should, for increased confidence, capture (1) the biological basis of the model structure and parameters; (2) performance of the model comparison and model simulations with experimental data; and (3) reliability of model predictions of dose metrics relevant to risk assessment (i.e., model testing, uncertainty, and sensitivity analyses).

### Children

**Fig. 5:**
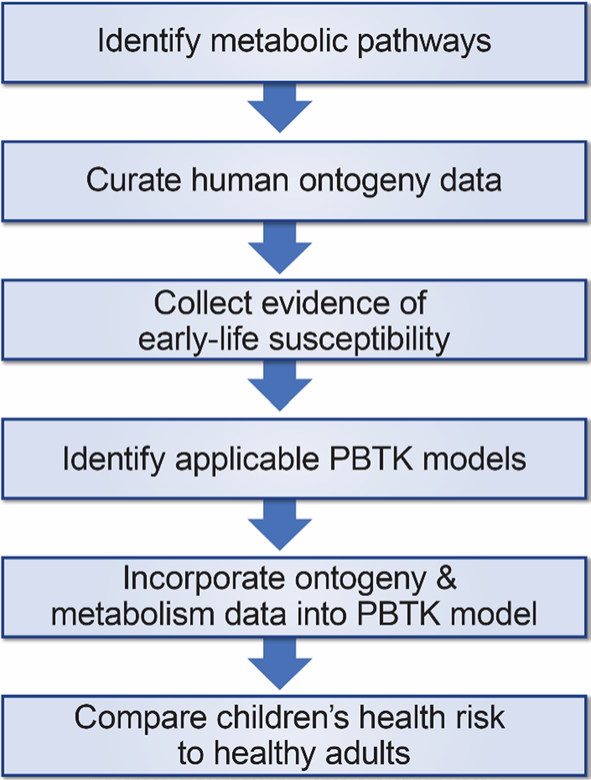
Approach for incorporating TK variability for children into risk assessment, adapted from Ginsberg et al. [7]

Children provide perhaps the most frequently assessed case of TK variability and its incorporation into human health risk evaluation. As a more consistent approach than simply deriving CSAFs “where such data exists,” Ginsberg et al. outlined a framework to guide risk assessments for childhood chemical exposures. In the paper outlining this approach, the ontogeny of metabolizing systems with chemical-specific exposures is used to compare child versus adult internal dose for five case study chemicals [9]. This approach is outlined in Fig. 5.

Tiered testing strategies have been proposed to identify chemicals for further prioritization and making decisions on which populations to target for risk assessment [23]. The approach outlined by Ginsberg et al. could be incorporated into a tiered testing strategy, and a similar strategy might be developed for other subpopulations and chemical groups.

### Drug development considerations

Approaches used in the pharmaceutical realm can provide additional examples for assessment and integration of TK variability that could be harnessed. In the drug safety arena, the U.S. FDA has been utilizing PK information and modeling to support drug safety for a number of years [11, 147]. The FDA accepts PBTK models to inform drug and biologics applications for potential waiving of clinical PK data [148]. Much of the current trend at the FDA is focused on predictive models, using quantitative structure activity relationships (QSARs) and PBTK models, such as for predicting drug-related toxicities, including drug-drug interactions, falling in line with the trend in the pharmaceutical industry. In 1991, roughly 40% of drug candidate attrition was due to ADME concerns. With increased use of ADME predictions and PBTK modeling, in 2000 that number dropped to 9% of all failures [149]. PK modeling can be used to predict therapeutic doses for certain populations, such as pediatrics, or other understudied patient populations. PBTK modeling on pediatric populations has received particular attention from stakeholders and is becoming commonplace in Phase I trials since companies are now required to submit data on pediatrics beforehand [92]. PBTK modeling has been used to determine dosing for children for numerous drugs, such as dolutegravir in neonates [150, 151]. In 2018 the FDA issued draft guidance for inclusion of pregnant women in clinical trials [152]. Similar PK modeling approaches have been used for evaluating variability brought on by diseases, such as renal impairment [42, 94, 153].

PBTK modeling has also allowed for estimating the contribution of multiple variables, enabling clinicians to adjust dosages for populations that might be more or less sensitive, contributing to model-informed precision dosing [154]. This approach combines PK and PD models with patient-specific data to individualize drug dosing. It aims to optimize the balance between efficacy and toxicity based on patient-specific criteria. However, widespread adoption of precision dosing has not occurred, remaining localized in certain academic centers. Adoption has been limited due to various challenges, including lack of needed background knowledge on the part of clinicians, a dearth of genotypic data, and cost/benefit analyses that limit interest on the part of hospitals and insurance.

## State of the science: PBTK models for variability evaluation

Providing greater specificity for certain subpopulations or chemicals of interest, PBTK models have been developed for application to scenarios of particular regulatory interest, such as pregnancy, per- and polyfluoroalkyl substances (PFAS), and mixtures. Case studies for each of these are discussed below.

### Time-varying parameters and models to evaluate pregnancy and gestation

Pregnancy is a particularly complex life-stage for PBTK modeling, as many parameters that can typically be described as constant are time-varying [155, 156]. Moreover, components that are not present in adults need to be accounted for, particularly for the fetus, which has its own distinct compartments and physiology, such as blood flow through the ductus venosus and foramen ovale [156]. These physiological and anatomical changes may subsequently impact metabolism and clearance both in the mother and in the fetus [157]. Examination of TK parameters during pregnancy is particularly important as fetal development presents a critical window of susceptibility that could result in life-long effects [156]. Despite the importance of and significant interest in this life-stage, there is a major dearth of data as most drugs are “off-label” for pregnancy, as pregnant women are typically excluded from clinical trials [156, 158]. Because of this, there is a critical need for development of pregnancy-specific PBTK models. Both FDA and the European Medicines Agency have issued pregnancy exposure guidelines–the European Medicines Agency for monitoring exposures throughout pregnancy and the FDA for prospective exposure registries while supporting PK/PD modeling [157, 158].

The development of pregnancy PBTK models relies on large datasets of physiological and anatomical parameters to account for the time-varying nature of this information and a wide degree of interindividual variability [158]. A number of data curation studies have been published identifying time-varying parameters and where gaps exist in the literature [155, 156, 158, 158, 159]. From these reviews, algorithms have been generated that describe anatomical and physiological changes with gestational age. Anatomical and physiological changes typically start in the first trimester, peak in the second trimester, and then stay relatively constant through birth [157, 158]. Significant physiological changes are seen such as in body weight, cardiac output, protein binding, renal clearance, respiration, levels of sex hormones, and intrauterine volumes [156–159]. Differences in TK properties, e.g., f_up_ and Cl_int_, are seen as well [155]. However, data are still sparse for certain parameters such as hepatic blood flow, early fetal growth, and metabolic enzyme activity, making it difficult to determine how these might be altered during pregnancy. Clinical observations of certain drugs support the observed physiological changes, such as increases in plasma volume resulting in increased V_d_ and reductions in plasma protein levels leading to increased f_up_ [159].

Algorithms from such data curation studies have been used to generate pregnancy PBTK models. The PBTK models can be further refined by including separate fetal compartments and/or considering the lactation phase [133, 155, 158, 160]. Comparisons of model predictions with in vivo experimental data demonstrate that these models provide reasonable estimates of concentrations, despite a high degree of uncertainty [155, 158]. Zhang et al. performed a sensitivity analysis to quantify how fetoplacental metabolism, gestational age, and placental transport impact fetal drug exposure following maternal drug exposure [133]. The umbilical venous: maternal plasma ratio, a common measure of fetal drug exposure, was found to be a poor indicator of fetal exposure except for under relatively steady-state conditions. A time-embedding network model underscored the importance of enzyme ontogeny—changes in enzyme expression—to chemical kinetics and toxicity [161]. This time-embedding model incorporated time-varying expression profiles for 23 enzymes, metabolites, and glutathione reactivity based on data on Americans eight weeks gestation to 18 years old, to model population and life-stage variability. Unlike most models that include a fixed degree of population variability, this time-embedding model allowed for capturing changes in variability over time based on the data. Such time-embedding models will allow for greater understanding of population and life-stage variability.

### Applying a pregnancy model for PFAS

Pregnancy PBTK models have been further applied to specific chemicals, such as PFAS [160]. PFAS are a class of chemicals that have been widely used for their chemical stability and properties, such as water- and oil-repellence and are thus found in a wide range of products ranging from cookware to food wrappers and furniture coatings [160, 162]. PFAS have garnered major public interest and concern in the past several years due to their widespread prevalence in humans—PFAS are detected in the blood of > 99% of individuals in the U.S.—and potential health effects: PFAS have been linked to developmental effects and alterations in lipids and immune function [160, 162]. Due to their chemical stability, PFAS typically have long half-lives in humans—over 5 years for some longer chain PFAS [153, 160, 162–164]. This contrasts with relatively short half-lives in rats, necessitating the importance of human-specific data [162]. The mounting concern over PFAS has led to the phasing out of several classes of PFAS, particularly perfluorooctanoic acid (PFOA) and perfluorooctane sulfonic acid (PFOS) since ~ 2000, correlating with a drop in serum levels since then [160, 162]. While PFAS are detected in nearly all individuals, there is a huge degree of interindividual variability, which can be largely attributed to geographic-specific exposures [162, 165, 166].

Variability in PFAS levels is particularly evident in pregnant and postpartum mothers due to several factors, such as parity, breastfeeding, age, and body mass index (BMI) [160]. Prior pregnancies and breastfeeding reduce the body burden of PFAS by increasing the V_d_ and preferential partitioning of PFAS into lipophilic tissues, including breast milk. To better understand fetal PFAS exposure, Brochot et al. developed a pregnancy and lactation PBTK model to estimate PFOA and PFOS in mothers and placental transfer to the fetus [160]. The model was built using PFAS exposure estimates derived from INMA, a prospective birth cohort study in Spain for which maternal blood spots were taken in either the first or second trimester and umbilical cord blood at delivery. The model was based on a generic maternal PBTK model to which a previously published fetal model was fit. Oral absorption was described through food and drinking water with elimination via breastfeeding and excretion, as well as blood loss during delivery. A dynamic exposure model was used to capture the drop in PFAS exposure beginning in ~ 2000. This model allowed for characterizing tissue distributions of PFOS and PFOA and showed differences in tissue distributions for the two compounds that fit with their distinct physicochemical properties. Moreover, the model showed reliability and broader applicability as it produced estimates that correlate well with levels reported in the U.S. population.

More general PBTK models for PFAS have been built as well, with many of these based on the U.S. population as this was the initial site of PFAS production [167]. Worley et al. developed and evaluated a PBTK model that incorporated renal transport mechanisms using IVIVE and MC to simulate serum PFOA concentrations following exposure to PFAS at levels reported in drinking water. Biomonitoring data were collected from individuals residing in two former major PFAS manufacturing sites, Ohio and Alabama, before and after filtration systems in public water systems were introduced, which brought PFAS water levels to below the limit of detection [162]. Although the model calibration revealed excellent agreement between the measured serum PFOA data and the predicted values, model evaluation with the biomonitoring samples noted an underestimation of the serum concentrations for a few of the communities, indicating that variability in PFAS exposure through non-drinking water sources may be involved. Additional MC simulations of inter-individual variability and variability in exposure (e.g., variations in drinking water source, distance from PFAS source) confirmed that non-drinking water sources may have a greater impact on exposures than previously thought.

Additional models exist as well. Chou et al. developed a multi-species model that was later refined as a Bayesian dose–response model to refine TK and TD uncertainties, as well as allow for performing population level probabilistic risk assessment [168]. Indeed, EFSA has used several such PFAS-specific PBTK models in their assessments of PFAS [169]. These compound-specific models provide more refined estimates than a generic PBTK model can, which can be important for particular chemicals of concern [170, 171].

### Lifetime exposure modeling

Lifetime exposure modeling tends to estimate cumulative exposure to a chemical over the course of a person's lifetime. By integrating data from various sources and considering the different factors influencing exposure, lifetime exposure modeling can provide a comprehensive assessment of the potential health risks associated with long-term exposure to a chemical, as well as assess the effect of exposure during critical time windows in late-life diagnosis pathologic endpoints.

Verner et al. developed a lifetime PBTK model to estimate lifetime blood/tissue exposure levels of persistent organic pollutants during hypothesized time windows of susceptibility in breast cancer development to assess the association between persistent organic pollutants and breast cancer incidence [172]. In the PBTK model, the values of physiologic parameters (e.g., organ volume, composition, and blood flow) throughout a woman’s entire life were estimated based on data on pregnancies, height, weight, and age. The lifetime TK profile for various exposure scenarios and physiologic factors (i.e., breastfeeding, growth, pregnancy, lactation, and body weight changes) was assessed. The study revealed that lactation periods and body weight changes are the factors that had the greatest impact on lifetime TK profiles.

In another study, a PBTK model was used to simulate blood levels of polychlorinated biphenyls during specific pre- and postnatal periods, which helped evaluate the association of chemical exposure with impairment of infant behaviors (e.g., attention, activity). Specific windows of susceptibility to individual infant behaviors were identified, highlighting the importance of modeling TK profiles during these periods (e.g., within the first year of life) [173].

When considering TK variability due to lifetime exposure, in addition to those factors discussed above, factors such as occupation, lifestyle, geographic location, and specific windows of susceptibility need to be included. The consideration of these additional factors shall reduce uncertainty linked to past chemical exposure and help to identify the impact of exposure during sensitive time windows to disease.

### Mixtures

Perhaps the greatest risk assessment challenge is adequate consideration of mixtures or chemical co-exposures and downstream effects. Indeed, the range of co-exposure scenarios is infinite and exposures can also occur via multiple routes. Often, study formulation is driven by understanding the impact of chemicals likely to interact with one another. PBTK models provide a useful framework to evaluate how TK can be impacted by chemical interactions. Examples are provided below to build our understanding of the impact of mixtures on TK and TD which may in turn stimulate thinking for future application.

Chemical-chemical interactions are impacted primarily by dose, administration route, and the number of compounds. Interactions can influence TK by impacting transport, binding properties, biotransformation, or formation of complexes that may impact physicochemical properties such as lipophilicity [171]. Mixture interactions can be modeled if all binary interactions are known, to give a network of interactions, though this becomes exponentially more complicated when more components are added to the system [171]. To simplify the process, it is suggested that only the interactions that most impact kinetics are modeled, and chemicals with similar physicochemical properties are lumped together. Alternatively, models that capture only specific pathways or for specific chemical groups can be modeled on their own.

Perhaps the most common example in pharmacology of how chemical interactions can influence human TK is that of CYP3A4 and grapefruit juice—an inhibitor of CYP3A4 that also inhibits the efflux transporter P-gp. Conversely, St. John’s wort is a commonly used inducer of CYP3A4 and P-gp. Being a major concern for pharmacology, Quignot et al. developed a meta-regression model based on an extensive literature search on the degree of interaction—or TK modulation—between CYP3A4 and P-gp substrates and grapefruit juice or St. John’s wort, as well as the degree of variability in metabolism [174]. The degree of interaction in kinetics (e.g., maximal concentration, AUC) was calculated based on the ratio of the binary mixture over the substrate alone. Substrate bioavailability and fraction metabolized were found to be the greatest contributors to the degree of interactions. To characterize variability, UFs were calculated for bioavailability and fraction metabolized, as well as the degree of interaction. For single compounds, UFs were all below the default. For multiple compounds, however, UFs reached 18.9 for acute exposure, and 17.1 for chronic exposure, when capturing the lowest bioavailability to highest fraction metabolized, in the presence of grapefruit juice.

A common example of mixtures of environmental compounds is pesticides, particularly pyrethroids. Quindroit et al. developed a PBTK model based on reverse dosimetry to estimate exposures to four pyrethroids—deltamethrin, permethrin, cypermethrin, and cyfluthrin, based on levels of metabolites measured in biomonitoring studies as these compounds produce several of the same metabolites and are metabolized by common pathways [175]. Effects of the individual pyrethroids were combined by dose addition, as this has been well-characterized in the literature, and interindividual variability in metabolism was assessed. Even with the incorporation of the default UF for interindividual variability, exposures were found to fall below a threshold of concern covering the 95th percentile.

Several PBTK models have been developed to model exposures to mixtures of volatile compounds and estimate human TK interindividual variability. Volatile compounds are not as well-characterized and provide additional complications but have provided insight into how exposure to mixtures can impact human TK variability at different life-stages, and the types of interactions that may occur [176, 177]. Co-exposures to drinking water contaminants, including benzene, trichloroethylene, and toluene, impacted the variability index for high exposures, with only minimal impact from low exposures. Variability generally fell below the default UF apart from high multi-route exposures at early life-stages. Sensitivity analyses, in which uncertainty and the influence of different model parameters are estimated, can be particularly informative for these complex models to determine drivers of variability. A sensitivity analysis for this model showed the blood:air partition coefficient to be the main driver of variability in the interaction model for the CYP2E1 pathway. For inhalation exposures to a similar set of compounds, TK variability was impacted based on the size of exposure, subpopulation, and substance type [177]. The number of compounds in the model did not matter at the low exposure level (20 ppm). At the high exposure level (50 ppm), the only interaction effect seen was in pregnant women where an increase in the number of compounds resulted in an increase in the maximal concentration of benzene. More sophisticated models must be developed to more comprehensively cover the range of chemical mixtures humans are exposed to, but these models provide a starting point.

## Conclusion: barriers to adoption and opportunities to advance the field

Incorporation of NAMs with PBTK modeling has allowed for making great strides in estimating variability for a range of sophisticated exposure scenarios, from evaluations of specific life-stages or diseased populations to cumulative exposures, lifetime exposures, or a combination thereof [11, 29, 120, 121, 147]. Data gaps remain, particularly for establishment of more comprehensive databases that capture ontogeny data for any and all enzymes and transporters to support chemical risk assessment. Significant progress was made for enzyme ontogeny data gathered over 15 years ago, with databases containing experimental ontogeny data [9, 40, 65, 178] and in vivo clinical PK data to support model evaluation [144]. One such database comprised information for 6 polymorphic enzymes—CYP2D6, CYP2E1, ALDH2, PON1, GSTs, and NATs, chosen based on knowledge of toxicity, genotype-phenotype, and linkage to environmentally-mediated disease to allow evaluation of how polymorphism can affect metabolism [12]. Although databases exist that capture physiologic parameters and population variability across typically healthy Caucasian populations, such data are largely absent for ethnic and diseased populations and specific life-stages requiring protection. Efforts to establish open-source databases of physiological parameters haven’t proven sustainable long-term thus far [95, 179]. Fortunately, a recent effort made great strides in capturing time-varying anatomical and physiological features for use in a maternal–fetal PBTK model [155, 156].

Including information on substrate turnover, how polymorphisms influence turnover, mode of action, and population variability in enzyme function allows for estimation of CSAFs, with certain enzymes and populations flagged that may warrant further consideration given values exceeding the default UFs. As more transporter and enzyme ontogeny data have been captured since then, an update and re-evaluation of existing data is warranted.

Despite progress to date, regulatory adoption of CSAFs for decision-making is still quite limited, although barriers to implementation have been identified [63]. A fundamental need is a call to modelers to provide transparent, explicit documentation of their model framework, parameterization and code to ensure model evaluation is possible. With the release of recent OECD guidance regarding PBTK model development along with several reports advocating for best practices in PBTK model reporting and ongoing stakeholder support for outreach and communication, these efforts should work to align subject matter experts with regulators grappling with interpreting modeling findings for regulatory application.

PBTK modeling has been demonstrated to be a powerful tool to evaluate interindividual and population TK and TD variability. With the advent of NAMs, many efforts further demonstrate its potential when applied with in vitro data streams, particularly for its ability to translate in vitro data out to in vivo relevant equivalent dosages. These applications have garnered much support from regulatory stakeholders, at least for certain decision-making needs [27, 138, 180]. As more sophisticated NAMs are developed, for instance microphysiological models to inform tissue-level responses, TK NAMs and models are poised to inform target tissue dosimetry. Although some gaps and barriers do remain that limit the ability to capture variability across all populations and life-stages that require consideration in risk assessment, research activities addressing these limitations are already underway, paving the way for future progress.

## Data Availability

No datasets were generated or analysed during the current study.

## Abbreviations

AC50: Activity concentration exerting 50% of maximum response
ADME: Absorption, distribution, metabolism, excretion
AUC: Area under the curve
BER: Bioactivity: exposure ratio
Cl_int_: Intrinsic clearance
CSAF: Chemical-specific adjustment factor
C_ss_: Steady-state concentration
CYP: Cytochrome P450
EPA: United States environmental protection agency
FDA: United States food & drug administration
f_up_: Fraction unbound in plasma
hiPSCs: Human induced pluripotent stem cells
HK_AF_: Human toxicokinetic adjustment factor
HTTK: High-throughput toxicokinetic
IVIVE: In vitro-in vivo extrapolation
MC: Monte Carlo
NAMs: New approach methodologies
NHANES: National health and nutrition examination survey
NRC: National research council
OECD: Organisation for economic cooperation and development
PBTK: Physiologically based toxicokinetic
PD: Pharmacodynamic
PFAS: Per- and polyfluoroalkyl substances
P-gp: P-glycoprotein
PK: Pharmacokinetic
QSAR: Quantitative structure activity relationship
TK: Toxicokinetic
TD: Toxicodynamic
UF: Uncertainty factor
UGT: Uridine 5'-diphospho-glucuronosyltransferase
V_d_: Volume of distribution

## References

[1] Li Y, Meng Q, Yang M, Liu D, Hou X, Tang L, et al. Current trends in drug metabolism and pharmacokinetics. Acta Pharm Sin B. 2019;9:1113–44. 10.1016/j.apsb.2019.10.001. 31867160 10.1016/j.apsb.2019.10.001 PMC6900561

[2] Bell SM, Chang X, Wambaugh JF, Allen DG, Bartels M, Brouwer KLR, et al. In vitro to in vivo extrapolation for high throughput prioritization and decision making. Toxicol Vitro. 2018;47:213–27. 10.1016/j.tiv.2017.11.016. 10.1016/j.tiv.2017.11.016 PMC7393693 29203341

[3] Bois FY, Jamei M, Clewell HJ. PBPK modelling of inter-individual variability in the pharmacokinetics of environmental chemicals. Toxicology. 2010;278:256–67. 10.1016/j.tox.2010.06.007. 20600548 10.1016/j.tox.2010.06.007

[4] Chiu WA, Barton HA, DeWoskin RS, Schlosser P, Thompson CM, Sonawane B, et al. Evaluation of physiologically based pharmacokinetic models for use in risk assessment. J Appl Toxicol. 2007;27:218–37. 10.1002/jat.1225. 17299829 10.1002/jat.1225

[5] Dorne JL, Renwick AG. The refinement of uncertainty/safety factors in risk assessment by the incorporation of data on toxicokinetic variability in humans. Toxicol Sci. 2005;86:20–6. 10.1093/toxsci/kfi160. 15800035 10.1093/toxsci/kfi160

[6] Andersen ME. Toxicokinetic modeling and its applications in chemical risk assessment. Toxicol Lett. 2003;138:9–27. 10.1016/s0378-4274(02)00375-2. 12559690 10.1016/s0378-4274(02)00375-2

[7] Lipscomb JC, Poet TS. In vitro measurements of metabolism for application in pharmacokinetic modeling. Pharmacol Ther. 2008;118:82–103. 10.1016/j.pharmthera.2008.01.006. 18374419 10.1016/j.pharmthera.2008.01.006

[8] Clewell HJ, Andersen ME. Use of physiologically based pharmacokinetic modeling to investigate individual versus population risk. Toxicology. 1996;111:315–29. 10.1016/0300-483X(96)03385-9. 8711746 10.1016/0300-483x(96)03385-9

[9] Ginsberg G, Vulimiri SV, Lin YS, Kancherla J, Foos B, Sonawane B. A framework and case studies for evaluation of enzyme ontogeny in children’s health risk evaluation. J Toxicol Environ Health A. 2017;80:569–93. 10.1080/15287394.2017.1369915. 28891786 10.1080/15287394.2017.1369915 PMC8018602

[10] Tracy TS, Chaudhry AS, Prasad B, Thummel KE, Schuetz EG, Zhong XB, et al. Interindividual variability in cytochrome P450-mediated drug metabolism. Drug Metab Dispos. 2016;44:343–51. 10.1124/dmd.115.067900. 26681736 10.1124/dmd.115.067900 PMC4767386

[11] Abdo N, Xia M, Brown CC, Kosyk O, Huang R, Sakamuru S, et al. Population-based in vitro hazard and concentration-response assessment of chemicals: the 1000 genomes high-throughput screening study. Environ Health Perspect. 2015;123:458–66. 10.1289/ehp.1408775. 25622337 10.1289/ehp.1408775 PMC4421772

[12] Ginsberg G, Smolenski S, Neafsey P, Hattis D, Walker K, Guyton KZ, et al. The influence of genetic polymorphisms on population variability in six xenobiotic-metabolizing enzymes. J Toxicol Environ Health B Crit Rev. 2009;12:307–33. 10.1080/10937400903158318. 20183525 10.1080/10937400903158318

[13] Hines RN. Ontogeny of human hepatic cytochromes P450. J Biochem Mol Toxicol. 2007;21:169–75. 10.1002/jbt.20179. 17936930 10.1002/jbt.20179

[14] Song G, Sun X, Hines RN, McCarver DG, Lake BG, Osimitz TG, et al. Determination of human hepatic CYP2C8 and CYP1A2 age-dependent expression to support human health risk assessment for early ages. Drug Metab Dispos. 2017;45:468–75. 10.1124/dmd.116.074583. 28228413 10.1124/dmd.116.074583

[15] Seripa D, Panza F, Daragjati J, Paroni G, Pilotto A. Measuring pharmacogenetics in special groups: geriatrics. Expert Opin Drug Metab Toxicol. 2015;11:1073–88. 10.1517/17425255.2015.1041919. 25990744 10.1517/17425255.2015.1041919

[16] Kiss M, Mbasu R, Nicolaï J, Barnouin K, Kotian A, Mooij MG, et al. Ontogeny of small intestinal drug transporters and metabolizing enzymes based on targeted quantitative proteomics. Drug Metab Dispos. 2021;49:1038–46. 10.1124/dmd.121.000559. 34548392 10.1124/dmd.121.000559

[17] van Groen BD, Allegaert K, Tibboel D, de Wildt SN. Innovative approaches and recent advances in the study of ontogeny of drug metabolism and transport. Br J Clin Pharmacol. 2022;88:4285–96. 10.1111/bcp.14534. 32851677 10.1111/bcp.14534 PMC9545189

[18] Sousa T, Paterson R, Moore V, Carlsson A, Abrahamsson B, Basit AW. The gastrointestinal microbiota as a site for the biotransformation of drugs. Int J Pharm. 2008;363:1–25. 10.1016/j.ijpharm.2008.07.009. 18682282 10.1016/j.ijpharm.2008.07.009

[19] Clarke G, Sandhu KV, Griffin BT, Dinan TG, Cryan JF, Hyland NP. Gut reactions: breaking down xenobiotic-microbiome interactions. Pharmacol Rev. 2019;71:198–224. 10.1124/pr.118.015768. 30890566 10.1124/pr.118.015768

[20] EFSA Scientific Committee, Hardy A, Benford D, Halldorsson T, Jeger MJ, Knutsen HK, et al. Guidance on the risk assessment of substances present in food intended for infants below 16 weeks of age. EFSA J. 2017;15:e04849. 10.2903/j.efsa.2017.4849. 32625502 10.2903/j.efsa.2017.4849 PMC7010120

[21] Loiodice S, da Nogueira Costa A, Atienzar F. Current trends in in silico, in vitro toxicology, and safety biomarkers in early drug development. Drug Chem Toxicol. 2019;42:113–21. 10.1080/01480545.2017.1400044. 29161932 10.1080/01480545.2017.1400044

[22] Kavlock RJ, Bahadori T, Barton-Maclaren TS, Gwinn MR, Rasenberg M, Thomas RS. Accelerating the pace of chemical risk assessment. Chem Res Toxicol. 2018;31:287–90. 10.1021/acs.chemrestox.7b00339. 29600706 10.1021/acs.chemrestox.7b00339 PMC6666390

[23] Thomas RS, Bahadori T, Buckley TJ, Cowden J, Deisenroth C, Dionisio KL, et al. The next generation blueprint of computational toxicology at the U.S. environmental protection agency. Toxicol Sci. 2019;169:317–32. 10.1093/toxsci/kfz058. 30835285 10.1093/toxsci/kfz058 PMC6542711

[24] Faber CM. Through the looking glass: in vitro models for inhalation toxicology and interindividual variability in the airway. Appl in Vitro Toxicol. 2018;4:115–28. 10.1089/aivt.2018.0002. 31380467 10.1089/aivt.2018.0002 PMC6067645

[25] Zhang Y, Zhang N, Niu Z. Health risk assessment of trihalomethanes mixtures from daily water-related activities via multi-pathway exposure based on PBPK model. Ecotoxicol Environ Saf. 2018;163:427–35. 10.1016/j.ecoenv.2018.07.073. 30075445 10.1016/j.ecoenv.2018.07.073

[26] Dobreniecki S, Mendez E, Lowit A, Freudenrich TM, Wallace K, Carpenter A, et al. Integration of toxicodynamic and toxicokinetic new approach methods into a weight-of-evidence analysis for pesticide developmental neurotoxicity assessment: a case-study with DL- and L-glufosinate. Regul Toxicol Pharmacol. 2022;131: 105167. 10.1016/j.yrtph.2022.105167. 35413399 10.1016/j.yrtph.2022.105167

[27] Health Canada. Science approach document–bioactivity exposure ratio: application in priority setting and risk assessment n.d.

[28] Chang X, Tan Y-M, Allen DG, Bell S, Brown PC, Browning L, et al. IVIVE: facilitating the use of in vitro toxicity data in risk assessment and decision making. Toxics. 2022;10:232. 10.3390/toxics10050232. 35622645 10.3390/toxics10050232 PMC9143724

[29] Wetmore BA, Allen B, Clewell HJ, Parker T, Wambaugh JF, Almond LM, et al. Incorporating population variability and susceptible subpopulations into dosimetry for high-throughput toxicity testing. Toxicol Sci. 2014;142:210–24. 10.1093/toxsci/kfu169. 25145659 10.1093/toxsci/kfu169

[30] Ginsberg G, Hattis D, Russ A, Sonawane B. Pharmacokinetic and pharmacodynamic factors that can affect sensitivity to neurotoxic sequelae in elderly individuals. Environ Health Perspect. 2005;113:1243–9. 10.1289/ehp.7568. 16140636 10.1289/ehp.7568 PMC1280410

[31] Gary G, William S, James B, Babasaheb S. Incorporating children’s toxicokinetics into a risk framework. Environ Health Perspect. 2004;112:272–83. 10.1289/ehp.6013. 14754583 10.1289/ehp.6013 PMC1241838

[32] Liang X, Feswick A, Simmons D, Martyniuk CJ. Environmental toxicology and omics: a question of sex. J Proteomics. 2018;172:152–64. 10.1016/j.jprot.2017.09.010. 29037750 10.1016/j.jprot.2017.09.010

[33] Abduljalil K, Johnson TN, Rostami-Hodjegan A. Fetal physiologically-based pharmacokinetic models: systems information on fetal biometry and gross composition. Clin Pharmacokinet. 2018;57:1149–71. 10.1007/s40262-017-0618-1. 29264787 10.1007/s40262-017-0618-1

[34] Craft NE, Haitema TB, Garnett KM, Fitch KA, Dorey CK. Carotenoid, tocopherol, and retinol concentrations in elderly human brain. J Nutr Health Aging. 2004;8:156–62. 15129301

[35] Kearns GL, Abdel-Rahman SM, Alander SW, Blowey DL, Leeder JS, Kauffman RE. Developmental pharmacology–drug disposition, action, and therapy in infants and children. N Engl J Med. 2003;349:1157–67. 10.1056/NEJMra035092. 13679531 10.1056/NEJMra035092

[36] Lu H, Rosenbaum S. Developmental pharmacokinetics in pediatric populations. J Pediatr Pharmacol Ther. 2014;19:262–76. 10.5863/1551-6776-19.4.262. 25762871 10.5863/1551-6776-19.4.262 PMC4341411

[37] Goasdoué K, Miller SM, Colditz PB, Björkman ST. Review: The blood-brain barrier; protecting the developing fetal brain. Placenta. 2017;54:111–6. 10.1016/j.placenta.2016.12.005. 27939102 10.1016/j.placenta.2016.12.005

[38] Ghersi-Egea J-F, Saudrais E, Strazielle N. Barriers to drug distribution into the perinatal and postnatal brain. Pharm Res. 2018;35:84. 10.1007/s11095-018-2375-8. 29516182 10.1007/s11095-018-2375-8

[39] Saili KS, Zurlinden TJ, Schwab AJ, Silvin A, Baker NC, Hunter ES 3rd, et al. Blood-brain barrier development: systems modeling and predictive toxicology. Birth Defects Res. 2017;109:1680–710. 10.1002/bdr2.1180. 29251840 10.1002/bdr2.1180 PMC6476421

[40] Ginsberg G. Pediatric pharmacokinetic data: implications for environmental risk assessment for children. Pediatrics. 2004;113:973–83. 15060190

[41] Gabarrón M, Faz A, Acosta JA. Soil or dust for health risk assessment studies in urban environment. Arch Environ Contam Toxicol. 2017;73:442–55. 10.1007/s00244-017-0413-x. 28528420 10.1007/s00244-017-0413-x

[42] Murphy WA, Adiwidjaja J, Sjöstedt N, Yang K, Beaudoin JJ, Spires J, et al. Considerations for physiologically based modeling in liver disease: from nonalcoholic fatty liver (NAFL) to nonalcoholic steatohepatitis (NASH). Clin Pharmacol Ther. 2023;113:275–97. 10.1002/cpt.2614. 35429164 10.1002/cpt.2614 PMC10083989

[43] Johnson TN, Boussery K, Rowland-Yeo K, Tucker GT, Rostami-Hodjegan A. A semi-mechanistic model to predict the effects of liver cirrhosis on drug clearance. Clin Pharmacokinet. 2010;49:189–206. 10.2165/11318160-000000000-00000. 20170207 10.2165/11318160-000000000-00000

[44] El-Khateeb E, Achour B, Al-Majdoub ZM, Barber J, Rostami-Hodjegan A. Non-uniformity of changes in drug-metabolizing enzymes and transporters in liver cirrhosis: implications for drug dosage adjustment. Mol Pharm. 2021;18:3563–77. 10.1021/acs.molpharmaceut.1c00462. 34428046 10.1021/acs.molpharmaceut.1c00462 PMC8424631

[45] Dadson P, Ferrannini E, Landini L, Hannukainen JC, Kalliokoski KK, Vaittinen M, et al. Fatty acid uptake and blood flow in adipose tissue compartments of morbidly obese subjects with or without type 2 diabetes: effects of bariatric surgery. Am J Physiol Endocrinol Metab. 2017;313:E175–82. 10.1152/ajpendo.00044.2017. 28400411 10.1152/ajpendo.00044.2017

[46] Bruno CD, Greenblatt DJ, Harmatz JS, Chow CR. Clinical consequences of altered drug disposition in obesity: call for change. J Clin Pharmacol. 2023;63(Suppl 2):S25-34. 10.1002/jcph.2308. 37942910 10.1002/jcph.2308

[47] Berton M, Bettonte S, Stader F, Battegay M, Marzolini C. Repository describing the anatomical, physiological, and biological changes in an obese population to inform physiologically based pharmacokinetic models. Clin Pharmacokinet. 2022;61:1251–70. 10.1007/s40262-022-01132-3. 35699913 10.1007/s40262-022-01132-3 PMC9439993

[48] Lea-Henry TN, Carland JE, Stocker SL, Sevastos J, Roberts DM. Clinical pharmacokinetics in kidney disease: fundamental principles. Clin J Am Soc Nephrol. 2018;13:1085–95. 10.2215/CJN.00340118. 29934432 10.2215/CJN.00340118 PMC6032582

[49] Yoshida K, Sun B, Zhang L, Zhao P, Abernethy DR, Nolin TD, et al. Systematic and quantitative assessment of the effect of chronic kidney disease on CYP2D6 and CYP3A4/5. Clin Pharmacol Ther. 2016;100:75–87. 10.1002/cpt.337. 26800425 10.1002/cpt.337 PMC5024330

[50] Tan SPF, Scotcher D, Rostami-Hodjegan A, Galetin A. Effect of chronic kidney disease on the renal secretion via organic anion transporters 1/3: implications for physiologically-based pharmacokinetic modeling and dose adjustment. Clin Pharmacol Ther. 2022;112:643–52. 10.1002/cpt.2642. 35569107 10.1002/cpt.2642 PMC9540491

[51] Dunvald A-CD, Järvinen E, Mortensen C, Stage TB. Clinical and molecular perspectives on inflammation-mediated regulation of drug metabolism and transport. Clin Pharmacol Ther. 2022;112:277–90. 10.1002/cpt.2432. 34605009 10.1002/cpt.2432

[52] Ginsberg G, Guyton K, Johns D, Schimek J, Angle K, Sonawane B. Genetic polymorphism in metabolism and host defense enzymes: implications for human health risk assessment. Crit Rev Toxicol. 2010;40:575–619. 10.3109/10408441003742895. 20662711 10.3109/10408441003742895

[53] Hiemke C, Shams M. Phenotyping and genotyping of drug metabolism to guide pharmacotherapy in psychiatry. Curr Drug Deliv. 2013;10:46–53. 10.2174/1567201811310010008. 22998042 10.2174/1567201811310010008

[54] Bae JW, Oh KY, Yoon SJ, Shin HB, Jung EH, Cho CK, et al. Effects of CYP2D6 genetic polymorphism on the pharmacokinetics of metoclopramide. Arch Pharm Res. 2020;43:1207–13. 10.1007/s12272-020-01293-4. 33247397 10.1007/s12272-020-01293-4

[55] Gaedigk A, Sangkuhl K, Whirl-Carrillo M, Klein T, Leeder JS. Prediction of CYP2D6 phenotype from genotype across world populations. Genet Med. 2017;19:69–76. 10.1038/gim.2016.80. 27388693 10.1038/gim.2016.80 PMC5292679

[56] Darney K, Lautz LS, Béchaux C, Wiecek W, Testai E, Amzal B, et al. Human variability in polymorphic CYP2D6 metabolism: Implications for the risk assessment of chemicals in food and emerging designer drugs. Environ Int. 2021;156: 106760. 10.1016/j.envint.2021.106760. 34256299 10.1016/j.envint.2021.106760

[57] Bi YA, Lin J, Mathialagan S, Tylaska L, Callegari E, Rodrigues AD, et al. Role of hepatic organic anion transporter 2 in the pharmacokinetics of R- and S-Warfarin: in vitro studies and mechanistic evaluation. Mol Pharm. 2018;15:1284–95. 10.1021/acs.molpharmaceut.7b01108. 29433307 10.1021/acs.molpharmaceut.7b01108

[58] Neul C, Hofmann U, Schaeffeler E, Winter S, Klein K, Giacomini KM, et al. Characterization of cytochrome P450 (CYP) 2D6 drugs as substrates of human organic cation transporters and multidrug and toxin extrusion proteins. Br J Pharmacol. 2021;178:1459–74. 10.1111/bph.15370. 33434947 10.1111/bph.15370

[59] Lipscomb JC, Meek ME, Krishnan K, Kedderis GL, Clewell H, Haber L. Incorporation of pharmacokinetic and pharmacodynamic data into risk assessments. Toxicol Mech Methods. 2004;14:145–58. 10.1080/15376520490429382. 20021141 10.1080/15376520490429382

[60] Hiratsuka M. In vitro assessment of the allelic variants of cytochrome P450. Drug Metab Pharmacokinet. 2012;27:68–84. 10.2133/dmpk.dmpk-11-rv-090. 22041138 10.2133/dmpk.dmpk-11-rv-090

[61] Yeo KR, Jamei M, Rostami-Hodjegan A. Predicting drug-drug interactions: application of physiologically based pharmacokinetic models under a systems biology approach. Expert Rev Clin Pharmacol. 2013;6:143–57. 23473592 10.1586/ecp.13.4

[62] Cresteil T. Onset of xenobiotic metabolism in children: toxicological implications. Food Addit Contam. 1998;15(Suppl):45–51. 10.1080/02652039809374614. 9602911 10.1080/02652039809374614

[63] Bhat VS, Meek MEB, Valcke M, English C, Boobis A, Brown R. Evolution of chemical-specific adjustment factors (CSAF) based on recent international experience; increasing utility and facilitating regulatory acceptance. Crit Rev Toxicol. 2017;47:729–49. 10.1080/10408444.2017.1303818. 28681680 10.1080/10408444.2017.1303818

[64] Ginsberg G, Hattis D, Sonawane B. Incorporating pharmacokinetic differences between children and adults in assessing children’s risks to environmental toxicants. Toxicol Appl Pharmacol. 2004;198:164–83. 10.1016/j.taap.2003.10.010. 15236952 10.1016/j.taap.2003.10.010

[65] Hines RN. Developmental expression of drug metabolizing enzymes: impact on disposition in neonates and young children. Int J Pharm. 2013;452:3–7. 10.1016/j.ijpharm.2012.05.079. 22766445 10.1016/j.ijpharm.2012.05.079

[66] Basit A, Fan PW, Khojasteh SC, Murray BP, Smith BJ, Heyward S, et al. Comparison of tissue abundance of non-cytochrome p450 drug-metabolizing enzymes by quantitative proteomics between humans and laboratory animal species. Drug Metab Dispos. 2022;50:197–203. 10.1124/dmd.121.000774. 34969659 10.1124/dmd.121.000774

[67] Ahire D, Patel M, Deshmukh SV, Prasad B. Quantification of accurate composition and total abundance of homologous proteins by conserved-plus-surrogate peptide approach: quantification of UDP glucuronosyltransferases in human tissues. Drug Metab Dispos. 2023;51:285. 10.1124/dmd.122.001155. 36446609 10.1124/dmd.122.001155

[68] Hines RN, Simpson PM, McCarver DG. Age-dependent human hepatic carboxylesterase 1 (CES1) and carboxylesterase 2 (CES2) postnatal ontogeny. Drug Metab Dispos. 2016;44:959–66. 10.1124/dmd.115.068957. 26825642 10.1124/dmd.115.068957

[69] Schulte RR, Ho RH. Organic anion transporting polypeptides: emerging roles in cancer pharmacology. Mol Pharmacol. 2019;95:490. 10.1124/mol.118.114314. 30782852 10.1124/mol.118.114314 PMC6442320

[70] Fu T, Zeng S, Zheng Q, Zhu F. The important role of transporter structures in drug disposition, efficacy, and toxicity. Drug Metab Dispos. 2023;51:1316. 10.1124/dmd.123.001275. 37295948 10.1124/dmd.123.001275

[71] Elmorsi Y, Barber J, Rostami-Hodjegan A. Ontogeny of hepatic drug transporters and relevance to drugs used in pediatrics. Drug Metab Dispos. 2016;44:992–8. 10.1124/dmd.115.067801. 26712821 10.1124/dmd.115.067801

[72] Cheung KWK, van Groen BD, Burckart GJ, Zhang L, de Wildt SN, Huang S-M. Incorporating ontogeny in physiologically based pharmacokinetic modeling to improve pediatric drug development: what we know about developmental changes in membrane transporters. J Clin Pharmacol. 2019;59(Suppl 1):S56-69. 10.1002/jcph.1489. 31502692 10.1002/jcph.1489 PMC7408403

[73] Anoshchenko O, Prasad B, Neradugomma NK, Wang J, Mao Q, Unadkat JD. Gestational age-dependent abundance of human placental transporters as determined by quantitative targeted proteomics. Drug Metab Dispos. 2020;48:735–41. 10.1124/dmd.120.000067. 32591415 10.1124/dmd.120.000067 PMC7469251

[74] Miller GW, Jones DP. The nature of nurture: refining the definition of the exposome. Toxicol Sci. 2014;137:1–2. 10.1093/toxsci/kft251. 24213143 10.1093/toxsci/kft251 PMC3871934

[75] Abouir K, Samer CF, Gloor Y, Desmeules JA, Daali Y. Reviewing data integrated for PBPK model development to predict metabolic drug-drug interactions: shifting perspectives and emerging trends. Front Pharmacol. 2021;12: 708299. 10.3389/fphar.2021.708299. 34776945 10.3389/fphar.2021.708299 PMC8582169

[76] Pavanello S, Lotti M. Biological monitoring of carcinogens: current status and perspectives. Arch Toxicol. 2012;86:535–41. 10.1007/s00204-011-0793-z. 22159923 10.1007/s00204-011-0793-z

[77] Cohen Hubal EA, de Wet T, Du Toit L, Firestone MP, Ruchirawat M, van Engelen J, et al. Identifying important life stages for monitoring and assessing risks from exposures to environmental contaminants: results of a world health organization review. Regul Toxicol Pharmacol. 2014;69:113–24. 10.1016/j.yrtph.2013.09.008. 24099754 10.1016/j.yrtph.2013.09.008 PMC5355211

[78] Hall SD, Thummel KE, Watkins PB, Lown KS, Benet LZ, Paine MF, et al. Molecular and physical mechanisms of first-pass extraction. Drug Metab Dispos. 1999;27:161–6. 9929497

[79] Goodrich JK, Davenport ER, Beaumont M, Jackson MA, Knight R, Ober C, et al. Genetic determinants of the gut microbiome in UK twins. Cell Host Microbe. 2016;19:731–43. 10.1016/j.chom.2016.04.017. 27173935 10.1016/j.chom.2016.04.017 PMC4915943

[80] Rothschild D, Weissbrod O, Barkan E, Kurilshikov A, Korem T, Zeevi D, et al. Environment dominates over host genetics in shaping human gut microbiota. Nature. 2018;555:210–5. 10.1038/nature25973. 29489753 10.1038/nature25973

[81] Claus SP, Guillou H, Ellero-Simatos S. The gut microbiota: a major player in the toxicity of environmental pollutants? NPJ Biofilms Microbiomes. 2016;2:16003. 10.1038/npjbiofilms.2016.3. 28721242 10.1038/npjbiofilms.2016.3 PMC5515271

[82] Joly C, Gay-Quéheillard J, Léké A, Chardon K, Delanaud S, Bach V, et al. Impact of chronic exposure to low doses of chlorpyrifos on the intestinal microbiota in the simulator of the human intestinal microbial ecosystem (SHIME) and in the rat. Environ Sci Pollut Res Int. 2013;20:2726–34. 10.1007/s11356-012-1283-4. 23135753 10.1007/s11356-012-1283-4

[83] Alderete TL, Jones RB, Chen Z, Kim JS, Habre R, Lurmann F, et al. Exposure to traffic-related air pollution and the composition of the gut microbiota in overweight and obese adolescents. Environ Res. 2018;161:472–8. 10.1016/j.envres.2017.11.046. 29220800 10.1016/j.envres.2017.11.046 PMC5747978

[84] Javurek AB, Spollen WG, Johnson SA, Bivens NJ, Bromert KH, Givan SA, et al. Effects of exposure to bisphenol A and ethinyl estradiol on the gut microbiota of parents and their offspring in a rodent model. Gut Microbes. 2016;7:471–85. 10.1080/19490976.2016.1234657. 27624382 10.1080/19490976.2016.1234657 PMC5103659

[85] Resnik DB, MacDougall DR, Smith EM. Ethical dilemmas in protecting susceptible subpopulations from environmental health risks: liberty, utility, fairness, and accountability for reasonableness. Am J Bioeth. 2018;18:29–41. 10.1080/15265161.2017.1418922. 29466133 10.1080/15265161.2017.1418922 PMC5884073

[86] Wason SC, Smith TJ, Perry MJ, Levy JI. Using physiologically-based pharmacokinetic models to incorporate chemical and non-chemical stressors into cumulative risk assessment: a case study of pesticide exposures. Int J Environ Res Public Health. 2012;9:1971–83. 10.3390/ijerph9051971. 22754485 10.3390/ijerph9051971 PMC3386599

[87] Sexton K, Olden K, Johnson BL. “Environmental justice”: the central role of research in establishing a credible scientific foundation for informed decision making. Toxicol Ind Health. 1993;9:685–727. 10.1177/074823379300900504. 8184441 10.1177/074823379300900504

[88] Nwanaji-Enwerem JC, Jackson CL, Ottinger MA, Cardenas A, James KA, Malecki KM, et al. Adopting a “Compound” exposome approach in environmental aging biomarker research a call to action for advancing racial health equity. Environ Health Perspect. 2021;129:045001. 10.1289/EHP8392. 33822649 10.1289/EHP8392 PMC8043128

[89] Teorell T. Studies on the diffusion effect upon ionic distribution : II. Experiments on ionic accumulation. J Gen Physiol. 1937;21:107–22. 10.1085/jgp.21.1.107. 19873036 10.1085/jgp.21.1.107 PMC2141929

[90] Rodgers T, Rowland M. Mechanistic approaches to volume of distribution predictions: understanding the processes. Pharm Res. 2007;24:918–33. 10.1007/s11095-006-9210-3. 17372687 10.1007/s11095-006-9210-3

[91] Poulin P, Theil F-P. Prediction of pharmacokinetics prior to in vivo studies. 1. Mechanism-based prediction of volume of distribution. J Pharm Sci. 2002;91:129–56. 10.1002/jps.10005. 11782904 10.1002/jps.10005

[92] Jones H, Rowland-Yeo K. Basic concepts in physiologically based pharmacokinetic modeling in drug discovery and development. CPT Pharmacometrics Syst Pharmacol. 2013;2: e63. 10.1038/psp.2013.41. 23945604 10.1038/psp.2013.41 PMC3828005

[93] Upton RN, Foster DJ, Abuhelwa AY. An introduction to physiologically-based pharmacokinetic models. Paediatr Anaesth. 2016;26:1036–46. 10.1111/pan.12995. 27550716 10.1111/pan.12995

[94] Johnson TN, Rostami-Hodjegan A, Tucker GT. Prediction of the clearance of eleven drugs and associated variability in neonates, infants and children. Clin Pharmacokinet. 2006;45:931–56. 10.2165/00003088-200645090-00005. 16928154 10.2165/00003088-200645090-00005

[95] Thompson CM, Johns DO, Sonawane B, Barton HA, Hattis D, Tardif R, et al. Database for physiologically based pharmacokinetic (PBPK) modeling: physiological data for healthy and health-impaired elderly. J Toxicol Environ Health B Crit Rev. 2009;12:1–24. 10.1080/10937400802545060. 19117207 10.1080/10937400802545060

[96] McNally K, Cotton R, Hogg A, Loizou G. PopGen: a virtual human population generator. Toxicology. 2014;315:70–85. 10.1016/j.tox.2013.07.009. 23876857 10.1016/j.tox.2013.07.009

[97] Pearce RG, Setzer RW, Strope CL, Wambaugh JF, Sipes NS. httk: R package for high-throughput toxicokinetics. J Stat Softw. 2017;79:1–26. 10.18637/jss.v079.i04. 30220889 10.18637/jss.v079.i04 PMC6134854

[98] Jamei M, Dickinson GL, Rostami-Hodjegan A. A framework for assessing inter-individual variability in pharmacokinetics using virtual human populations and integrating general knowledge of physical chemistry, biology, anatomy, physiology and genetics: a tale of “bottom-up” vs “top-down” recognition of covariates. Drug Metab Pharmacokinet. 2009;24:53–75. 10.2133/dmpk.24.53. 19252336 10.2133/dmpk.24.53

[99] Ring CL, Pearce RG, Setzer RW, Wetmore BA, Wambaugh JF. Identifying populations sensitive to environmental chemicals by simulating toxicokinetic variability. Environ Int. 2017;106:105–18. 10.1016/j.envint.2017.06.004. 28628784 10.1016/j.envint.2017.06.004 PMC6116525

[100] Calvier EAM, Krekels EHJ, Johnson TN, Rostami-Hodjegan A, Tibboel D, Knibbe CAJ. Scaling drug clearance from adults to the young children for drugs undergoing hepatic metabolism: a simulation study to search for the simplest scaling method. AAPS J. 2019;21:38. 10.1208/s12248-019-0295-0. 30850923 10.1208/s12248-019-0295-0 PMC6505506

[101] Howgate EM, Rowland Yeo K, Proctor NJ, Tucker GT, Rostami-Hodjegan A. Prediction of in vivo drug clearance from in vitro data. I: impact of inter-individual variability. Xenobiotica. 2006;36:473–97. 10.1080/00498250600683197. 16769646 10.1080/00498250600683197

[102] Al-Subeihi AA, Alhusainy W, Kiwamoto R, Spenkelink B, van Bladeren PJ, Rietjens IM, et al. Evaluation of the interindividual human variation in bioactivation of methyleugenol using physiologically based kinetic modeling and Monte Carlo simulations. Toxicol Appl Pharmacol. 2015;283:117–26. 10.1016/j.taap.2014.12.009. 25549870 10.1016/j.taap.2014.12.009

[103] Strikwold M, Spenkelink B, Woutersen RA, Rietjens I, Punt A. Development of a combined in vitro physiologically based kinetic (PBK) and Monte Carlo modelling approach to predict interindividual human variation in phenol-induced developmental toxicity. Toxicol Sci. 2017;157:365–76. 10.1093/toxsci/kfx054. 28498972 10.1093/toxsci/kfx054

[104] Ghosh J, Lawless MS, Waldman M, Gombar V, Fraczkiewicz R. Modeling ADMET. In: Benfenati E, editor. In silico methods for predicting drug toxicity. New York: Springer; 2016. p. 63–83. 10.1007/978-1-4939-3609-0_4.

[105] Li Y, Shao W, Wang X, Geng K, Wang W, Liu Z, et al. Physiologically based pharmacokinetic model of brivaracetam to predict the exposure and dose exploration in hepatic impairment and elderly populations. J Pharm Sci. 2024;S0022–3549(24):00348–54. 10.1016/j.xphs.2024.08.022. 10.1016/j.xphs.2024.08.022 39243975

[106] Grzegorzewski J, Brandhorst J, König M. Physiologically based pharmacokinetic (PBPK) modeling of the role of CYP2D6 polymorphism for metabolic phenotyping with dextromethorphan. Front Pharmacol. 2022;13:1029073. 10.3389/fphar.2022.1029073. 36353484 10.3389/fphar.2022.1029073 PMC9637881

[107] Kenyon EM, Lipscomb JC, Pegram RA, George BJ, Hines RN. The impact of scaling factor variability on risk-relevant pharmacokinetic outcomes in children: A case study using bromodichloromethane (BDCM). Toxicol Sci. 2019;167:347–59. 10.1093/toxsci/kfy236. 30252107 10.1093/toxsci/kfy236 PMC10448349

[108] Waters NJ, Obach RS, Di L. Consideration of the unbound drug concentration in enzyme kinetics. In: Nagar S, Argikar UA, Tweedie D, editors. Enzyme kinetics in drug metabolism: fundamentals and applications. New York: Springer; 2021. p. 113–45. 10.1007/978-1-0716-1554-6_5.

[109] OECD. Guidance document on good in vitro method practices (GIVIMP), OECD Series on Testing and Assessment. Paris: OECD Publishing 2018.

[110] Gouliarmou V, Lostia AM, Coecke S, Bernasconi C, Bessems J, Dorne JL, et al. Establishing a systematic framework to characterise in vitro methods for human hepatic metabolic clearance. Toxicol In Vitro. 2018;53:233–44. 10.1016/j.tiv.2018.08.004. 30099088 10.1016/j.tiv.2018.08.004 PMC10288526

[111] Louisse J, de Jong E, van de Sandt JJM, Blaauboer BJ, Woutersen RA, Piersma AH, et al. The use of in vitro toxicity data and physiologically based kinetic modeling to predict dose-response curves for in vivo developmental toxicity of glycol ethers in rat and man. Toxicol Sci. 2010;118:470–84. 10.1093/toxsci/kfq270. 20833708 10.1093/toxsci/kfq270

[112] Kramer NI, Di Consiglio E, Blaauboer BJ, Testai E. Biokinetics in repeated-dosing in vitro drug toxicity studies. Toxicol In Vitro. 2015;30:217–24. 10.1016/j.tiv.2015.09.005. 26362508 10.1016/j.tiv.2015.09.005

[113] Proença S, Escher BI, Fischer FC, Fisher C, Grégoire S, Hewitt NJ, et al. Effective exposure of chemicals in *in vitro* cell systems: a review of chemical distribution models. Toxicol In Vitro. 2021;73: 105133. 10.1016/j.tiv.2021.105133. 33662518 10.1016/j.tiv.2021.105133

[114] Nicol B, Vandenbossche-Goddard E, Thorpe C, Newman R, Patel H, Yates D. A workflow to practically apply true dose considerations to *in vitro* testing for next generation risk assessment. Toxicology. 2024;505: 153826. 10.1016/j.tox.2024.153826. 38719068 10.1016/j.tox.2024.153826

[115] Armitage JM, Wania F, Arnot JA. Application of mass balance models and the chemical activity concept to facilitate the use of in vitro toxicity data for risk assessment. Environ Sci Technol. 2014;48:9770–9. 10.1021/es501955g. 25014875 10.1021/es501955g

[116] Huchthausen J, Mühlenbrink M, König M, Escher BI, Henneberger L. Experimental exposure assessment of ionizable organic chemicals in in vitro cell-based bioassays. Chem Res Toxicol. 2020;33:1845–54. 10.1021/acs.chemrestox.0c00067. 32368900 10.1021/acs.chemrestox.0c00067

[117] Wetmore BA, Wambaugh JF, Ferguson SS, Sochaski MA, Rotroff DM, Freeman K, et al. Integration of dosimetry, exposure, and high-throughput screening data in chemical toxicity assessment. Toxicol Sci. 2012;125:157–74. 10.1093/toxsci/kfr254. 21948869 10.1093/toxsci/kfr254

[118] Wetmore BA, Wambaugh JF, Allen B, Ferguson SS, Sochaski MA, Setzer RW, et al. Incorporating high-throughput exposure predictions with dosimetry-adjusted in vitro bioactivity to inform chemical toxicity testing. Toxicol Sci. 2015;148:121–36. 10.1093/toxsci/kfv171. 26251325 10.1093/toxsci/kfv171 PMC4620046

[119] Wetmore BA, Wambaugh JF, Ferguson SS, Li L, Clewell HJ, Judson RS, et al. Relative impact of incorporating pharmacokinetics on predicting in vivo hazard and mode of action from high-throughput in vitro toxicity assays. Toxicol Sci. 2013;132:327–46. 10.1093/toxsci/kft012. 23358191 10.1093/toxsci/kft012

[120] Zhang Q, Li J, Middleton A, Bhattacharya S, Conolly RB. Bridging the data gap from in vitro toxicity testing to chemical safety assessment through computational modeling. Front Public Health. 2018;6:261. 10.3389/fpubh.2018.00261. 30255008 10.3389/fpubh.2018.00261 PMC6141783

[121] Burnett SD, Blanchette AD, Grimm FA, House JS, Reif DM, Wright FA, et al. Population-based toxicity screening in human induced pluripotent stem cell-derived cardiomyocytes. Toxicol Appl Pharmacol. 2019;381: 114711. 10.1016/j.taap.2019.114711. 31425687 10.1016/j.taap.2019.114711 PMC6745256

[122] Costin GE, Raabe HA. Optimized in vitro pigmentation screening assay using a reconstructed three dimensional human skin model. Rom J Biochem. 2013;50:15–27.

[123] Sun H, Chow EC, Liu S, Du Y, Pang KS. The caco-2 cell monolayer: usefulness and limitations. Expert Opin Drug Metab Toxicol. 2008;4:395–411. 10.1517/17425255.4.4.395. 18433344 10.1517/17425255.4.4.395

[124] Ahluwalia N, Herrick K, Paulose-Ram R, Johnson C. Data needs for B-24 and beyond: NHANES data relevant for nutrition surveillance of infants and young children. Am J Clin Nutr. 2014;99:747S-S754. 10.3945/ajcn.113.069062. 24452232 10.3945/ajcn.113.069062 PMC6331057

[125] Wambaugh JF, Wang A, Dionisio KL, Frame A, Egeghy P, Judson R, et al. High throughput heuristics for prioritizing human exposure to environmental chemicals. Environ Sci Technol. 2014;48:12760–7. 10.1021/es503583j. 25343693 10.1021/es503583j

[126] Gaudenzio N. Height-dimensional profiling of immune response to injectable drugs using bio-stabilized natural human skin 2022.

[127] Carberry CK, Ferguson SS, Beltran AS, Fry RC, Rager JE. Using liver models generated from human-induced pluripotent stem cells (iPSCs) for evaluating chemical-induced modifications and disease across liver developmental stages. Toxicol In Vitro. 2022;83: 105412. 10.1016/j.tiv.2022.105412. 35688329 10.1016/j.tiv.2022.105412 PMC9296547

[128] Kasteel EEJ, Westerink RHS. Refining in vitro and in silico neurotoxicity approaches by accounting for interspecies and interindividual differences in toxicodynamics. Expert Opin Drug Metab Toxicol. 2021;17:1007–17. 10.1080/17425255.2021.1885647. 33586568 10.1080/17425255.2021.1885647

[129] Chetty M, Rose RH, Abduljalil K, Patel N, Lu G, Cain T, et al. Applications of linking PBPK and PD models to predict the impact of genotypic variability, formulation differences, differences in target binding capacity and target site drug concentrations on drug responses and variability. Front Pharmacol. 2014;5:258. 10.3389/fphar.2014.00258. 25505415 10.3389/fphar.2014.00258 PMC4244809

[130] Yang RS, Thomas RS, Gustafson DL, Campain J, Benjamin SA, Verhaar HJ, et al. Approaches to developing alternative and predictive toxicology based on PBPK/PD and QSAR modeling. Environ Health Perspect. 1998;106(Suppl 6):1385–93. 10.1289/ehp.98106s61385. 9860897 10.1289/ehp.98106s61385 PMC1533423

[131] Knaak JB, Dary CC, Zhang X, Gerlach RW, Tornero-Velez R, Chang DT, et al. Parameters for pyrethroid insecticide QSAR and PBPK/PD models for human risk assessment. Rev Environ Contam Toxicol. 2012;219:1–114. 10.1007/978-1-4614-3281-4_1. 22610175 10.1007/978-1-4614-3281-4_1

[132] Bi Y, Liu J, Li L, Yu J, Bhattaram A, Bewernitz M, et al. Role of model-informed drug development in pediatric drug development, regulatory evaluation, and labeling. J Clin Pharmacol. 2019;59(Suppl 1):S104–11. 10.1002/jcph.1478. 31502691 10.1002/jcph.1478

[133] Zhang Z, Imperial MZ, Patilea-Vrana GI, Wedagedera J, Gaohua L, Unadkat JD. Development of a novel maternal-fetal physiologically based pharmacokinetic model I: Insights into factors that determine fetal drug exposure through simulations and sensitivity analyses. Drug Metab Dispos. 2017;45:920–38. 10.1124/dmd.117.075192. 28588050 10.1124/dmd.117.075192 PMC5506457

[134] Krewski D, Acosta DJ, Andersen M, Anderson H, Bailar JC 3rd, Boekelheide K, et al. Toxicity testing in the 21st century: a vision and a strategy. J Toxicol Environ Health B Crit Rev. 2010;13:51–138. 10.1080/10937404.2010.483176. 20574894 10.1080/10937404.2010.483176 PMC4410863

[135] National Research Council. Science and decisions: advancing risk assessment. Washington, DC: The National Academies Press; 2009. 25009905

[136] Tonnelier A, Coecke S, Zaldívar J-M. Screening of chemicals for human bioaccumulative potential with a physiologically based toxicokinetic model. Arch Toxicol. 2012;86:393–403. 10.1007/s00204-011-0768-0. 22089525 10.1007/s00204-011-0768-0 PMC3282909

[137] Rotroff DM, Wetmore BA, Dix DJ, Ferguson SS, Clewell HJ, Houck KA, et al. Incorporating human dosimetry and exposure into high-throughput in vitro toxicity screening. Toxicol Sci. 2010;117:348–58. 10.1093/toxsci/kfq220. 20639261 10.1093/toxsci/kfq220 PMC6280841

[138] Paul Friedman K, Gagne M, Loo L-H, Karamertzanis P, Netzeva T, Sobanski T, et al. Utility of in vitro bioactivity as a lower bound estimate of in vivo adverse effect levels and in risk-based prioritization. Toxicol Sci. 2020;173:202–25. 10.1093/toxsci/kfz201. 31532525 10.1093/toxsci/kfz201 PMC7720780

[139] Dornbos P, LaPres JJ. Incorporating population-level genetic variability within laboratory models in toxicology: from the individual to the population. Toxicology. 2018;395:1–8. 10.1016/j.tox.2017.12.007. 29275117 10.1016/j.tox.2017.12.007 PMC5801153

[140] Renwick AG. Data-derived safety factors for the evaluation of food additives and environmental contaminants. Food Addit Contam. 1993;10:275–305. 10.1080/02652039309374152. 8359312 10.1080/02652039309374152

[141] Renwick AG, Lazarus NR. Human variability and noncancer risk assessment–an analysis of the default uncertainty factor. Regul Toxicol Pharmacol. 1998;27:3–20. 9618319 10.1006/rtph.1997.1195

[142] WHO/IPCS. Chemical-specific adjustment factors (CSAF) for interspecies differences and human variability: guidance document for the use of data in dose/concentration-response assessment. n.d.

[143] Dorne JLCM. Human variability in hepatic and renal elimination: implications for risk assessment. J Appl Toxicol. 2007;27:411–20. 10.1002/jat.1255. 17497760 10.1002/jat.1255

[144] Hattis D, Baird S, Goble R. A straw man proposal for a quantitative definition of the RfD. Drug Chem Toxicol. 2002;25:403–36. 10.1081/DCT-120014793. 12378950 10.1081/dct-120014793

[145] Abdo N, Wetmore BA, Chappell GA, Shea D, Wright FA, Rusyn I. In vitro screening for population variability in toxicity of pesticide-containing mixtures. Environ Int. 2015;85:147–55. 10.1016/j.envint.2015.09.012. 26386728 10.1016/j.envint.2015.09.012 PMC4773193

[146] Dorne JL. Metabolism, variability and risk assessment. Toxicology. 2010;268:156–64. 10.1016/j.tox.2009.11.004. 19932147 10.1016/j.tox.2009.11.004

[147] Rostami-Hodjegan A. Physiologically based pharmacokinetics joined with in vitro-in vivo extrapolation of ADME: a marriage under the arch of systems pharmacology. Clin Pharmacol Ther. 2012;92:50–61. 10.1038/clpt.2012.65. 22644330 10.1038/clpt.2012.65

[148] Food and Drug Administration C for DE and R, U. S. Department of Health and Human Services. Physiologically Based Pharmacokinetic Analyses — Format and Content Guidance for Industry. 2018.

[149] Kola I, John L. Can the pharmaceutical industry reduce attrition rates? Nat Rev Drug Discovery. 2004;3:711–6. 10.1038/nrd1470. 15286737 10.1038/nrd1470

[150] Bunglawala F, Rajoli RKR, Mirochnick M, Owen A, Siccardi M. Prediction of dolutegravir pharmacokinetics and dose optimization in neonates via physiologically based pharmacokinetic (PBPK) modelling. J Antimicrob Chemother. 2020;75:640–7. 10.1093/jac/dkz506. 31860112 10.1093/jac/dkz506

[151] Wang K, Jiang K, Wei X, Li Y, Wang T, Song Y. Physiologically based pharmacokinetic models are effective support for pediatric drug development. AAPS PharmSciTech. 2021;22:208. 10.1208/s12249-021-02076-w. 34312742 10.1208/s12249-021-02076-w PMC8312709

[152] Shields KE. FDA draft guidance-pregnant women: scientific and ethical considerations for inclusion in clinical trials. Pregnancy and the pharmaceutical industry: the movement towards evidence-based pharmacotherapy for pregnant women 2019. p. 183–97.

[153] Younis IR, Robert Powell J, Rostami-Hodjegan A, Corrigan B, Stockbridge N, Sinha V, et al. Utility of model-based approaches for informing dosing recommendations in specific populations: report from the public AAPS workshop. J Clin Pharmacol. 2017;57:105–9. 10.1002/jcph.787. 27365151 10.1002/jcph.787

[154] Darwich AS, Ogungbenro K, Vinks AA, Powell JR, Reny JL, Marsousi N, et al. Why has model-informed precision dosing not yet become common clinical reality? Lessons from the past and a roadmap for the future. Clin Pharmacol Ther. 2017;101:646–56. 10.1002/cpt.659. 28182269 10.1002/cpt.659

[155] Kapraun DF, Sfeir M, Pearce RG, Davidson-Fritz SE, Lumen A, Dallmann A, et al. Evaluation of a rapid, generic human gestational dose model. Reprod Toxicol. 2022;113:172–88. 10.1016/j.reprotox.2022.09.004. 36122840 10.1016/j.reprotox.2022.09.004 PMC9761697

[156] Kapraun DF, Wambaugh JF, Setzer RW, Judson RS. Empirical models for anatomical and physiological changes in a human mother and fetus during pregnancy and gestation. PLoS ONE. 2019;14: e0215906. 10.1371/journal.pone.0215906. 31048866 10.1371/journal.pone.0215906 PMC6497258

[157] Abduljalil K, Furness P, Johnson TN, Rostami-Hodjegan A, Soltani H. Anatomical, physiological and metabolic changes with gestational age during normal pregnancy: a database for parameters required in physiologically based pharmacokinetic modelling. Clin Pharmacokinet. 2012;51:365–96. 10.2165/11597440-000000000-00000. 22515555 10.2165/11597440-000000000-00000

[158] Gaohua L, Abduljalil K, Jamei M, Johnson TN, Rostami-Hodjegan A. A pregnancy physiologically based pharmacokinetic (p-PBPK) model for disposition of drugs metabolized by CYP1A2, CYP2D6 and CYP3A4. Br J Clin Pharmacol. 2012;74:873–85. 10.1111/j.1365-2125.2012.04363.x. 22725721 10.1111/j.1365-2125.2012.04363.x PMC3495152

[159] Dallmann A, Ince I, Meyer M, Willmann S, Eissing T, Hempel G. Gestation-specific changes in the anatomy and physiology of healthy pregnant women: an extended repository of model parameters for physiologically based pharmacokinetic modeling in pregnancy. Clin Pharmacokinet. 2017;56:1303–30. 10.1007/s40262-017-0539-z. 28401479 10.1007/s40262-017-0539-z

[160] Brochot C, Casas M, Manzano-Salgado C, Zeman FA, Schettgen T, Vrijheid M, et al. Prediction of maternal and foetal exposures to perfluoroalkyl compounds in a Spanish birth cohort using toxicokinetic modelling. Toxicol Appl Pharmacol. 2019;379: 114640. 10.1016/j.taap.2019.114640. 31251942 10.1016/j.taap.2019.114640

[161] Matlock MK, Tambe A, Elliott-Higgins J, Hines RN, Miller GP, Swamidass SJ. A time-embedding network models the ontogeny of 23 hepatic drug metabolizing enzymes. Chem Res Toxicol. 2019;32:1707–21. 10.1021/acs.chemrestox.9b00223. 31304741 10.1021/acs.chemrestox.9b00223 PMC6933754

[162] Worley RR, Yang X, Fisher J. Physiologically based pharmacokinetic modeling of human exposure to perfluorooctanoic acid suggests historical non drinking-water exposures are important for predicting current serum concentrations. Toxicol Appl Pharmacol. 2017;330:9–21. 10.1016/j.taap.2017.07.001. 28684146 10.1016/j.taap.2017.07.001 PMC5664934

[163] Pizzurro DM, Seeley M, Kerper LE, Beck BD. Interspecies differences in perfluoroalkyl substances (PFAS) toxicokinetics and application to health-based criteria. Regul Toxicol Pharmacol. 2019;106:239–50. 10.1016/j.yrtph.2019.05.008. 31078680 10.1016/j.yrtph.2019.05.008

[164] Fàbrega F, Kumar V, Benfenati E, Schuhmacher M, Domingo JL, Nadal M. Physiologically based pharmacokinetic modeling of perfluoroalkyl substances in the human body. Toxicol Environ Chem. 2015;97:814–27. 10.1080/02772248.2015.1060976.

[165] Bartolome M, Gallego-Pico A, Cutanda F, Huetos O, Esteban M, Perez-Gomez B, et al. Perfluorinated alkyl substances in Spanish adults: geographical distribution and determinants of exposure. Sci Total Environ. 2017;603–604:352–60. 10.1016/j.scitotenv.2017.06.031. 28633112 10.1016/j.scitotenv.2017.06.031

[166] Evich MG, Davis MJB, McCord JP, Acrey B, Awkerman JA, Knappe DRU, et al. Per- and polyfluoroalkyl substances in the environment. Science. 2022;375:eabg9065. 10.1126/science.abg9065. 35113710 10.1126/science.abg9065 PMC8902460

[167] East A, Dawson DE, Brady S, Vallero DA, Tornero-Velez R. A scoping assessment of implemented toxicokinetic models of per- and Polyfluoro-Alkyl substances, with a focus on one-compartment models. Toxics. 2023. 10.3390/toxics11020163. 36851038 10.3390/toxics11020163 PMC9964825

[168] Chou W-C, Lin Z. Probabilistic human health risk assessment of perfluorooctane sulfonate (PFOS) by integrating in vitro, in vivo toxicity, and human epidemiological studies using a Bayesian-based dose-response assessment coupled with physiologically based pharmacokinetic (PBPK) modeling approach. Environ Int. 2020;137: 105581. 10.1016/j.envint.2020.105581. 32087483 10.1016/j.envint.2020.105581

[169] EFSA Panel on Contaminants in the Food Chain (EFSA CONTAM Panel), Schrenk D, Bignami M, Bodin L, Chipman JK, Del Mazo J, et al. Risk to human health related to the presence of perfluoroalkyl substances in food. EFSA J. 2020;18:e06223. 10.2903/j.efsa.2020.6223. 32994824 10.2903/j.efsa.2020.6223 PMC7507523

[170] Lave T, Parrott N, Grimm HP, Fleury A, Reddy M. Challenges and opportunities with modelling and simulation in drug discovery and drug development. Xenobiotica. 2007;37:1295–310. 10.1080/00498250701534885. 17968746 10.1080/00498250701534885

[171] Haddad S, Poulin P, Funk C. Extrapolating in vitro metabolic interactions to isolated perfused liver: predictions of metabolic interactions between R-bufuralol, bunitrolol, and debrisoquine. J Pharm Sci. 2010;99:4406–26. 10.1002/jps.22136. 20310018 10.1002/jps.22136

[172] Verner M-A, Charbonneau M, López-Carrillo L, Haddad S. Physiologically based pharmacokinetic modeling of persistent organic pollutants for lifetime exposure assessment: a new tool in breast cancer epidemiologic studies. Environ Health Perspect. 2008;116:886–92. 10.1289/ehp.10917. 18629310 10.1289/ehp.10917 PMC2453156

[173] Verner M-A, Ayotte P, Muckle G, Charbonneau M, Haddad S. A physiologically based pharmacokinetic model for the assessment of infant exposure to persistent organic pollutants in epidemiologic studies. Environ Health Perspect. 2009;117:481–7. 10.1289/ehp.0800047. 19337526 10.1289/ehp.0800047 PMC2661921

[174] Quignot N, Wiecek W, Amzal B, Dorne JL. The Yin-Yang of CYP3A4: a Bayesian meta-analysis to quantify inhibition and induction of CYP3A4 metabolism in humans and refine uncertainty factors for mixture risk assessment. Arch Toxicol. 2019;93:107–19. 10.1007/s00204-018-2325-6. 30298208 10.1007/s00204-018-2325-6

[175] Quindroit P, Crepet A, Brochot C. Estimating human exposure to pyrethroids’ mixtures from biomonitoring data using physiologically based pharmacokinetic modeling. Environ Res. 2021;192: 110281. 10.1016/j.envres.2020.110281. 33031810 10.1016/j.envres.2020.110281

[176] Tohon H, Valcke M, Haddad S. An assessment of the impact of multi-route co-exposures on human variability in toxicokinetics: a case study with binary and quaternary mixtures of volatile drinking water contaminants. J Appl Toxicol. 2019;39:974–91. 10.1002/jat.3787. 30834571 10.1002/jat.3787

[177] Valcke M, Haddad S. Assessing human variability in kinetics for exposures to multiple environmental chemicals: a physiologically based pharmacokinetic modeling case study with dichloromethane, benzene, toluene, ethylbenzene, and m-xylene. J Toxicol Environ Health A. 2015;78:409–31. 10.1080/15287394.2014.971477. 25785556 10.1080/15287394.2014.971477

[178] McCarver DG, Simpson PM, Kocarek TA, James MO, Runge-Morris Me, Stevens JC, Yoon M, Hines RN. Data from: Developmental expression of drug metabolizing enzymes: impact on disposition in neonates and young children n.d. 10.1016/j.ijpharm.2012.05.079 22766445

[179] Brown RP, Delp MD, Lindstedt SL, Rhomberg LR, Beliles RP. Physiological parameter values for physiologically based pharmacokinetic models. Toxicol Ind Health. 1997;13:407–84. 10.1177/074823379701300401. 9249929 10.1177/074823379701300401

[180] Using 21st Century Science to Improve Risk-Related Evaluations. Washington (DC): 2017. 10.17226/24635. 28267305

